# Bufalin Inhibits the PI3K/AKT Pathway by Targeting GTF3C4 to Impede Breast Cancer Progression

**DOI:** 10.1002/advs.202507008

**Published:** 2026-02-06

**Authors:** Siyu Guo, Xiaodong Chen, Haojia Wang, Jiying Zhou, Peiying Lu, Jiangying Liu, Keyan Chai, Jingyuan Zhang, Siyun Yang, Shan Lu, Yifei Gao, Zhengsen Jin, Xiaoyu Tao, Zhongdong Hu, Qinglin Li, Jiarui Wu

**Affiliations:** ^1^ Department of Clinical Chinese Pharmacy School of Chinese Materia Medica Beijing University of Chinese Medicine Beijing China; ^2^ Modern Research Center For Traditional Chinese Medicine Beijing Institute of Traditional Chinese Medicine Beijing University of Chinese Medicine Beijing China; ^3^ Hangzhou Institute of Medicine (HIM) Zhejiang Cancer Hospital Chinese Academy of Sciences Hangzhou Zhejiang China

**Keywords:** breast cancer, Bufalin, GTF3C4, PI3K/AKT pathway, tumor microenvironment

## Abstract

Breast cancer incidence is rising globally, presenting challenges such as treatment side effects and drug resistance. Bufalin is a bufadienolides compound with potential anti‐cancer effects. This study shows that bufalin inhibits malignant proliferation of MDA‐MB‐231 and MCF‐7 cells and protects mice against breast cancer. Of note, GTF3C4 was identified as the target protein by Limited Proteolysis‐Mass Spectrometry. GTF3C4 is overexpressed in breast cancer and associated with poor prognosis. RNA sequencing analysis reveals that the PI3K/AKT signaling pathway is a key contributor. Using cell thermal shift assays, drug affinity response target stability assays, and surface plasmon resonance, it was verified that bufalin can specifically bind to GTF3C4. Bufalin reduces GTF3C4 protein levels in vivo and in vitro, effectively inhibiting breast cancer progression by suppressing the PI3K/AKT signaling pathway. After the knockdown of GTF3C4, the PI3K/AKT signaling pathway is also suppressed, thereby inhibiting the proliferation of breast cancer cells and promoting apoptosis. Single‐cell RNA sequencing results indicated that bufalin reduces the proportions of macrophages, neutrophils, and monocytes, and affects the strength of receptor‐ligand signals between cells. Collectively, this study demonstrates that bufalin targets GTF3C4 to inhibit the PI3K/AKT pathway and remodels the tumor microenvironment, thereby hindering the malignant progression of breast cancer.

## Graphical abstract

Mechanisms of Bufalin‐Mediated Intervention in Breast Cancer.

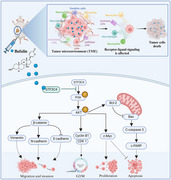

## 1 Introduction

Breast cancer has the highest incidence among malignant tumors in women, accounting for 32% of all female malignancie [1]. Despite improvements in the overall survival and prognosis of breast cancer patients in recent years, oncologists still face significant challenges in reducing the incidence of breast cancer and preventing recurrence and metastasis [2]. Breast cancer is a highly heterogeneous malignancy, exhibiting significant morphological and molecular heterogeneity both between tumors and within individual tumors [3]. Therefore, there is an urgent need to explore new treatment strategies to enhance therapeutic efficacy and improve the quality of life for patients. In recent years, research into the molecular mechanisms of breast cancer has deepened, providing new insights for the development of novel targeted drugs [4, 5, 6]. A comprehensive understanding of the biological characteristics of breast cancer and the regulatory mechanisms of its microenvironment will not only enhance the effectiveness of existing treatments but may also provide new avenues for future therapies.

Bufonis venenum, a traditional natural medicine, has been used for treating different diseases [7]. Bufonis venenum is a source of lead compounds for the development of potential cancer treatment drugs [8, 9]. Bufalin, a bufadienolide compound in bufonis venenum, has garnered significant attention in the field of cancer therapy in recent years including lung cancer [10], breast cancer [11], colorectal cancer [12], and gastric cancer [13]. Notably, bufalin exhibits a range of antitumor properties, including the ability to inhibit tumor cell proliferation, induce apoptosis, and suppress tumor metastasis [14]. In breast cancer, bufalin has shown significant inhibitory effects on multiple breast cancer cell lines, with mechanisms that likely involve the regulation of various signaling pathways, including the RIP1/RIP3/PARP‐1 and RIP1/RIP3/PGAM5 pathways [11, 15]. Bufalin also inhibits the activities of steroid receptor co‐activators SRC‐3 and SRC‐1, induces ferroptosis in these cells by targeting the SLC7A11/GPX4 pathway regulated by DECR1, and reverses ABCB1‐mediated resistance to docetaxel in breast cancer treatment [16, 17, 18]. This evidence highlights the potential of bufalin as a promising therapeutic agent for the treatment of breast cancer. However, the studies on the binding target proteins of bufalin against breast cancer and its effects on the breast cancer tumor microenvironment remain unclear.

RNA sequencing (RNA‐seq) and single‐cell RNA sequencing (scRNA‐seq) are becoming increasingly essential in cancer research, offering powerful tools for a deeper understanding of the molecular mechanisms underlying the disease [19, 20]. RNA‐seq reveals key genes and signaling pathways associated with tumor progression, drug resistance, and metastasis by analyzing the expression levels of all RNA in cells. ScRNA‐seq further refines this perspective, allowing researchers to explore tumor heterogeneity at the single‐cell level and identify interactions between tumor cells and different cell types in the tumor microenvironment. This meticulous analysis helps identify new biomarkers and potential therapeutic targets. In addition, limited proteolysis‐mass spectrometry (LiP‐MS), as an emerging technology, can effectively identify target proteins bound to drugs and reveal the mechanism of action of drugs [21]. The combination of RNA‐seq, scRNA‐seq, and LiP‐MS provides a new perspective and method for the study and treatment of cancer.

In this study, we revealed the potential efficacy of bufalin in the treatment of breast cancer in cells and animal models. Utilizing LiP‐MS and RNA‐seq, we discovered that bufalin binds to the GTF3C4 and suppresses the PI3K/AKT pathway. Knockdown GTF3C4 inhibits the proliferation of breast cancer cells, promotes apoptosis, and inhibits the activation of PI3K/AKT signaling. Furthermore, we discovered that bufalin modulates the breast cancer tumor microenvironment by scRNA‐seq. Collectively, this study explored the mechanisms by which bufalin acts against breast cancer, highlighting the roles of GTF3C4 and PI3K/AKT pathway. The findings suggest that bufalin could hold significant promise for breast cancer therapy.

## 2 Results

### 2.1 The Inhibitory Effect of Bufalin on Malignant Behavior of MDA‐MB‐231 and MCF‐7 Cells

**FIGURE 1:**
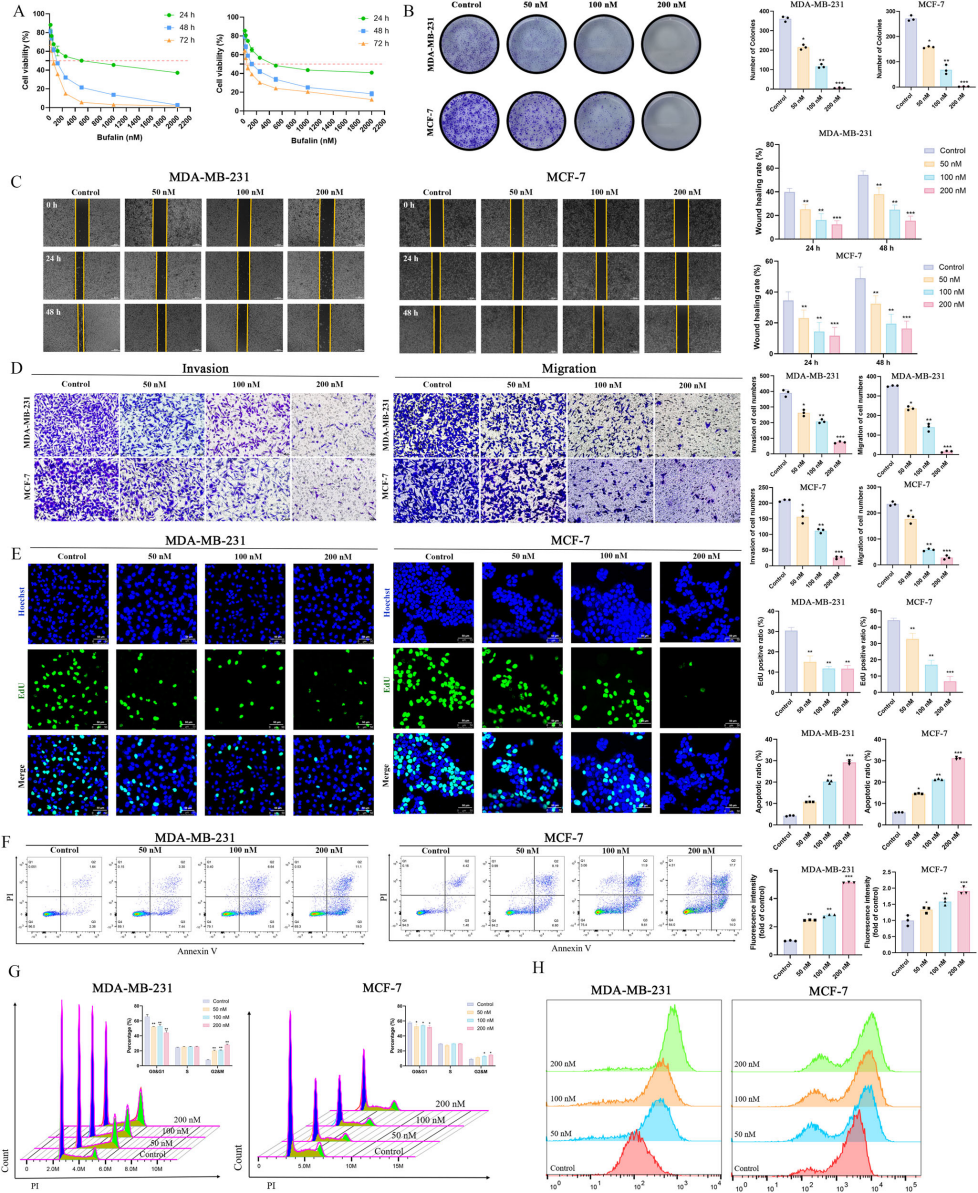
The inhibitory effect of bufalin on the malignant behavior of MDA‐MB‐231 and MCF‐7 cells. (A) Cell viability at 24, 48, and 72 h. (B) Cell colony formation. (C) Wound healing (500 µm). (D) Invasion and Migration (50 µm). (E) EdU cell proliferation. (F) Apoptosis. (G) Cell cycle distribution. (H) Reactive oxygen species. (*n* = 3, ^*^P < 0.05, ^**^P < 0.01 and ^***^P < 0.001).

The effect of bufalin on the proliferation of breast cancer cells MDA‐MB‐231 and MCF‐7 was explored by CCK‐8 assay. Bufalin inhibited the proliferation of MDA‐MB‐231 and MCF‐7 cells in both a dose‐dependent and time‐dependent manner. The IC_50_ values of bufalin for MDA‐MB‐231 cell viability at 24, 48, and 72 h were 500.83 ± 25.52, 104.77 ± 5.26, and 53.01 ± 2.75 nm, respectively. For MCF‐7 cells, the IC_50_ values at 24, 48, and 72 h were 582.30 ± 15.40, 136.60 ± 19.38, and 50.14 ± 7.98 nm, respectively (Figure 1A). As the concentration of bufalin increased, the number of MDA‐MB‐231 and MCF‐7 cell clones progressively decreased (Figure 1B). The rate of wound healing significantly declined with higher concentrations of bufalin (Figure 1C). Bufalin also reduced the migration and invasion of MDA‐MB‐231 and MCF‐7 cells (Figure 1D). In Figure 1E, bufalin reduced the proportion of EdU‐positive cells in both MDA‐MB‐231 and MCF‐7 cell lines. After 48 h of treatment with bufalin, apoptosis was significantly induced in MDA‐MB‐231 and MCF‐7 cells (Figure 1F). The flow cytometry results indicated that bufalin decreased the proportion of MDA‐MB‐231 and MCF‐7 cells in the G0/G1 phase, and increased the proportion in the G2/M phase (Figure 1G). In addition, bufalin increased reactive oxygen species (ROS) accumulation in MDA‐MB‐231 and MCF‐7 cells. The accumulation of reactive oxygen species may enhance the antitumor effects of bufalin (Figure 1H). Collectively, these data demonstrate that bufalin exerts potent anti‐proliferative, anti‐migratory, and pro‐apoptotic effects in breast cancer cells, accompanied by cell cycle arrest and ROS accumulation, suggesting its multi‐faceted role in suppressing tumor progression.

### 2.2 Bufalin Exerts Antitumor Efficacy In Vivo

**FIGURE 2:**
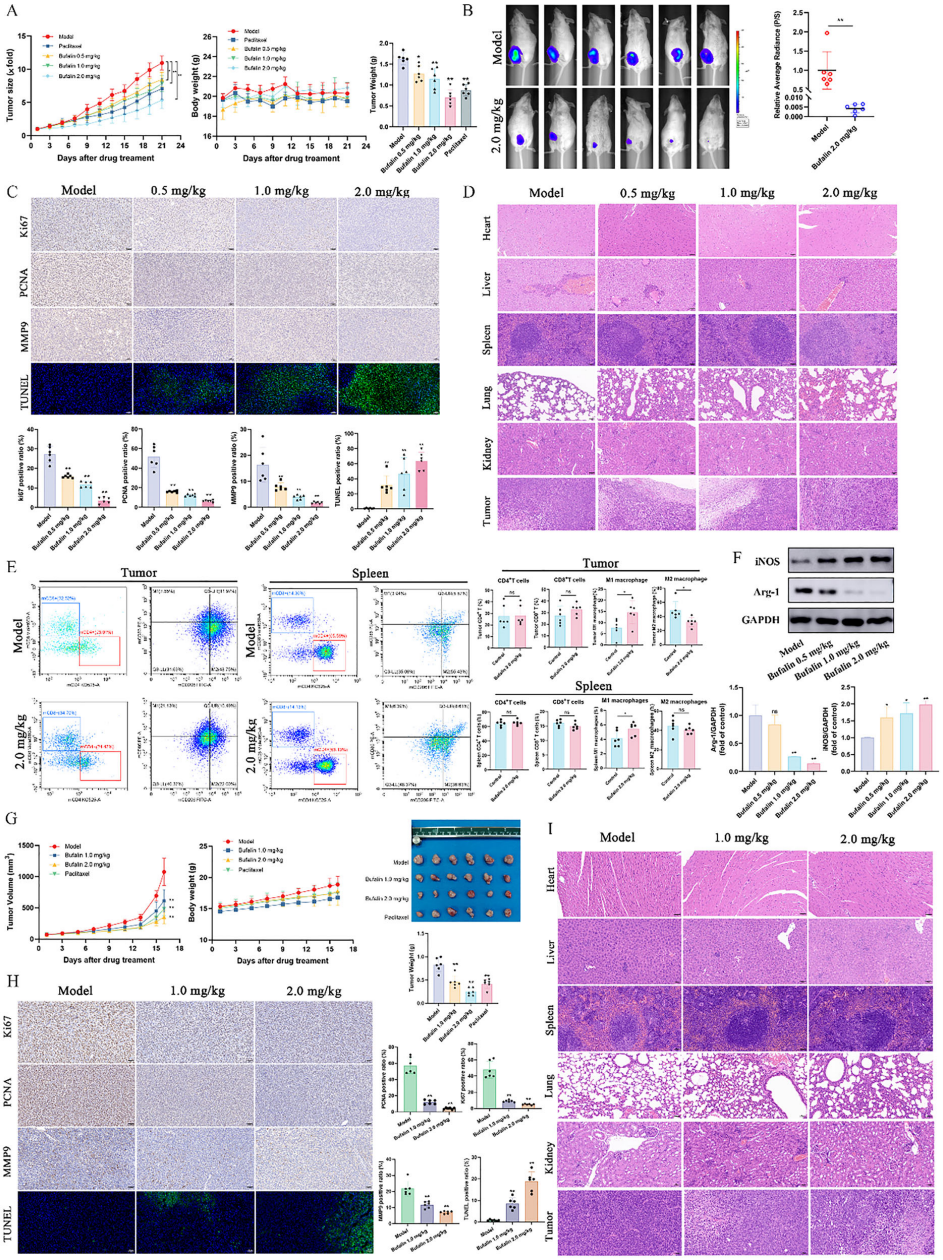
Bufalin exerts antitumor efficacy in vivo. (A) The changes in tumor size, body weight and tumor weight in mice using the 4T1‐luc model. (B) In vivo imaging. (C) Immunohistochemical analysis of Ki67, PCNA, and MMP9, along with TUNEL staining in tumor tissues of the 4T1‐luc model (50 µm). (D) H&E staining of the heart, liver, spleen, lung, kidney, and tumor tissues in the 4T1‐luc model (50 µm). (E) Flow cytometry of T cells and macrophages in the tumor and spleen of the 4T1‐luc model. (F) The expression levels of iNOS and Arg‐1 in tumors of the 4T1‐luc model (*n* = 3). (G) The changes in tumor size, body weight, image of tumor, and tumor weight in mice using the MDA‐MB‐231 model. (H) Immunohistochemical analysis of Ki67, PCNA, and MMP9, along with TUNEL staining in tumor tissues of the MDA‐MB‐231 model (50 µm). (I) H&E staining of the heart, liver, spleen, lung, kidney, and tumor tissues in the MDA‐MB‐231 model (50 µm). (*n* = 6, ^*^P < 0.05, ^**^P < 0.01 and ns represented no statistical difference).

To evaluate the in vivo anti‐breast cancer effect of bufalin, 4T1‐luc breast cancer cells were subcutaneously injected into BALB/c mice. MDA‐MB‐231 cells was used to construct a subcutaneous tumor transplantation model in BALB/c nude mice. Notably, bufalin could significantly inhibit the growth of tumors in mice compared with the model group, while there was no significant change in body weight (Figure 2A,G). In vivo imaging results demonstrated that bufalin at a dose of 2.0 mg/kg suppressed tumor growth in the 4T1‐luc  model (Figure 2B). Immunohistochemistry (IHC) staining displayed a meaningful diminution of Ki67, PCNA, and MMP9 positive cells in bufalin‐treated groups compared with the model group, suggesting that bufalin inhibited the proliferation and metastasis of tumor (Figure 2C,H). TUNEL staining results showed that the TUNEL‐positive cells were increased in the bufalin treatment groups, indicating that bufalin can induce apoptosis of tumor cells (Figure 2C,H). The histopathological examination of the mice did not reveal any apparent abnormalities (Figure 2D,I). In addition, there were no significant differences in alanine aminotransferase (ALT), aspartate aminotransferase (AST), blood urea nitrogen (BUN), uric acid (UA), lactate dehydrogenase (LDH), and creatine kinase (CK) levels among all groups (Figure S1A,B). These results indicate that bufalin possesses certain safety. In addition, flow cytometry was used to assess the levels of T cells and macrophages in the tumor and spleen tissues of mice in the 4T1‐luc model. As shown in Figure 2E, there were no significant differences in the proportions of CD4^+^ and CD8^+^ T cells in the tumor and spleen tissues. However, the proportion of M1 macrophages significantly increased, while M2 macrophages significantly decreased in the tumor tissues. In the spleen tissues, M1 macrophages were significantly increased. After bufalin treatment, the expression levels of iNOS were upregulated in tumors, while those of Arg‐1 were downregulated (Figure 2F). IHC further demonstrated that bufalin significantly upregulated the expression of CD86 while downregulating the expression of CD206 in tumor tissue (Figure S1C). These results suggested that bufalin has potential immunomodulatory effects in the breast cancer mouse model. Taken together, bufalin demonstrates potent anti‐tumor activity in two models by suppressing tumor proliferation, metastasis, and inducing apoptosis, while also modulating macrophage polarization towards an anti‐tumor M1 phenotype.

### 2.3 Bufalin Targets the GTF3C4 Protein and PI3K/AKT Signaling Pathways

**FIGURE 3:**
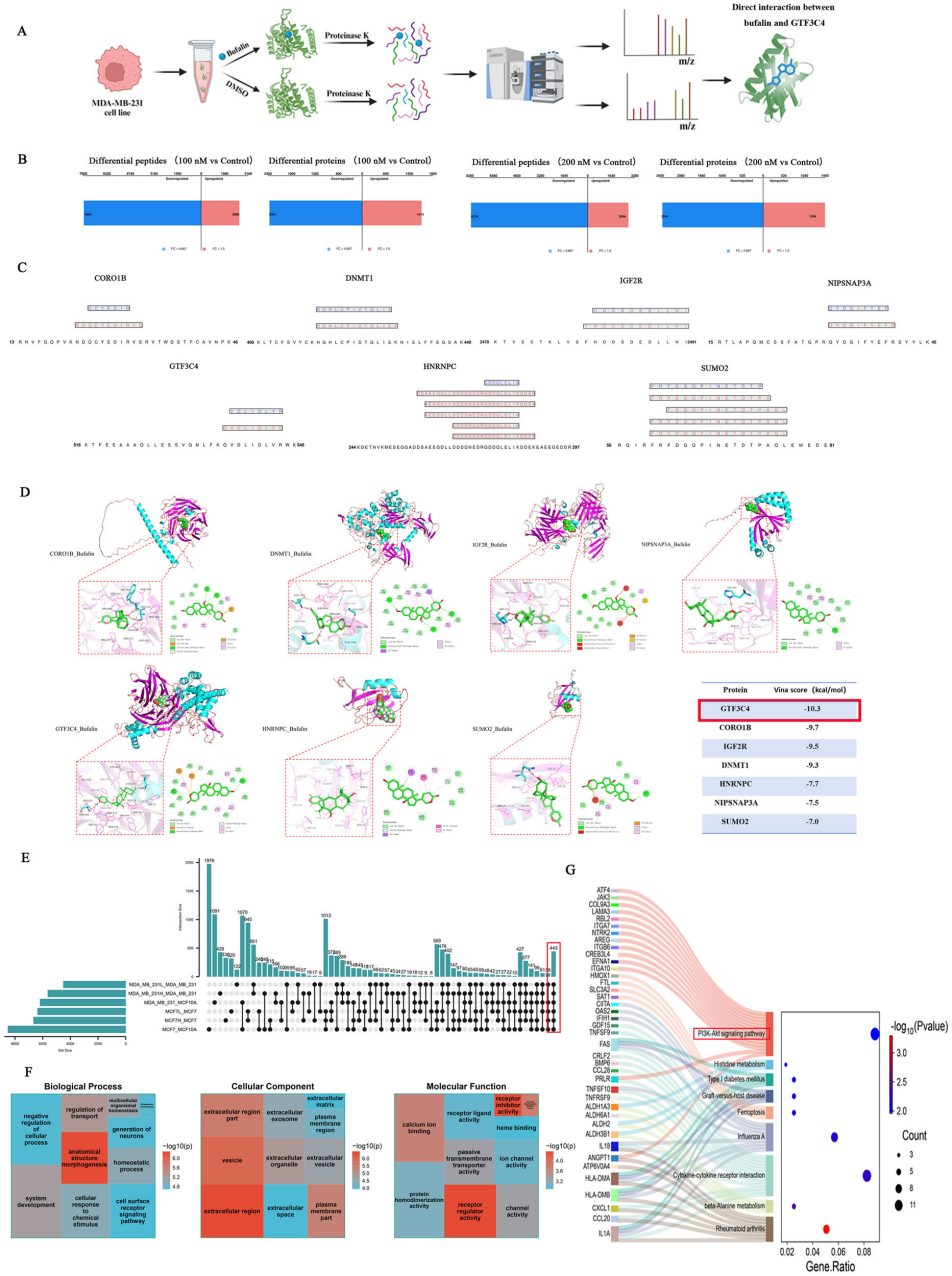
The targeting protein and signaling pathways of bufalin. (A) The flowchart of LiP‐MS. (B) Differential peptides and proteins (100 nm vs Control, 200 nm vs Control). (C) Peptides segment localization map. (D) Molecule docking was performed for bufalin with seven proteins (CORO1B, DNMT1, IGF2R, NIPSNAP3A, GTF3C4, HNRNPC, and SUMO2), and the corresponding Vina scores. (E) Screening of key genes abnormally expressed in breast cancer regulated by bufalin. (F,G) GO and KEGG enrichment analysis.

Bufalin exhibits strong anti‐breast cancer effects both in vitro and in vivo, yet the underlying molecular mechanism requires elucidation. To further elucidate the molecular mechanisms underlying these effects, we employed LiP‐MS and RNA‐seq to identify key target proteins and signaling pathways of bufalin in breast cancer. In this flowchart, MDA‐MB‐231‐derived cell lysates were incubated with bufalin or DMSO, followed by limited proteolysis with proteinase K, trypsin, and LC‐MS/MS (Figure 3A). The number of differences in peptides and proteins between BUF (100 and 200 nm) versus control were shown in Figure 3B (FC > 1.5 or FC < 0.667, and *p* < 0.05). The feature sequence analysis identified seven proteins as potential target proteins: CORO1B, DNMT1, IGF2R, NIPSNAP3A, GTF3C4, HNRNPC, and SUMO2 (Figure 3C). Molecular docking was performed between bufalin and the potential target proteins (Figure 3D). The docking score of bufalin with GTF3C4 is ‐10.3 kcal/mol, which is the lowest among all the docking scores. Bufalin formed hydrogen bonds with SER254 and VAL482 at the binding site of GTF3C4 (Figure 3D). This suggests that GTF3C4 has a relatively high potential to be a binding target protein for bufalin. RNA‐seq was conducted on MCF‐10A, MCF‐7, and MDA‐MB‐231 cells, as well as on MCF‐7 and MDA‐MB‐231 cells treated with bufalin, to further explore the key genes and pathways involved in the anti‐breast cancer effects of bufalin. To identify key genes modulating the aberrant expression of bufalin in breast cancer, we conducted differential gene expression analysis across multiple experimental groups and intersected the results (Figure S2). This integrative approach yielded a total of 443 candidate genes for further validation (Figure 3E). GO enrichment analysis was performed on 443 key genes. BP is mainly related to anatomical structure morphogenesis, regulation of transport, and homeostasis in multicellular organisms (Figure 3F). CC is mainly associated with extracellular regions, vesicles, and plasma membrane parts (Figure 3F). MF is mainly related to receptor regulatory activity, receptor inhibitor activity, and calcium ion binding (Figure 3F). The KEGG results showed that the PI3K/AKT signaling pathway was enriched with the most key genes (Figure 3G).

### 2.4 Bufalin Reshaped the Tumor Microenvironment

**FIGURE 4:**
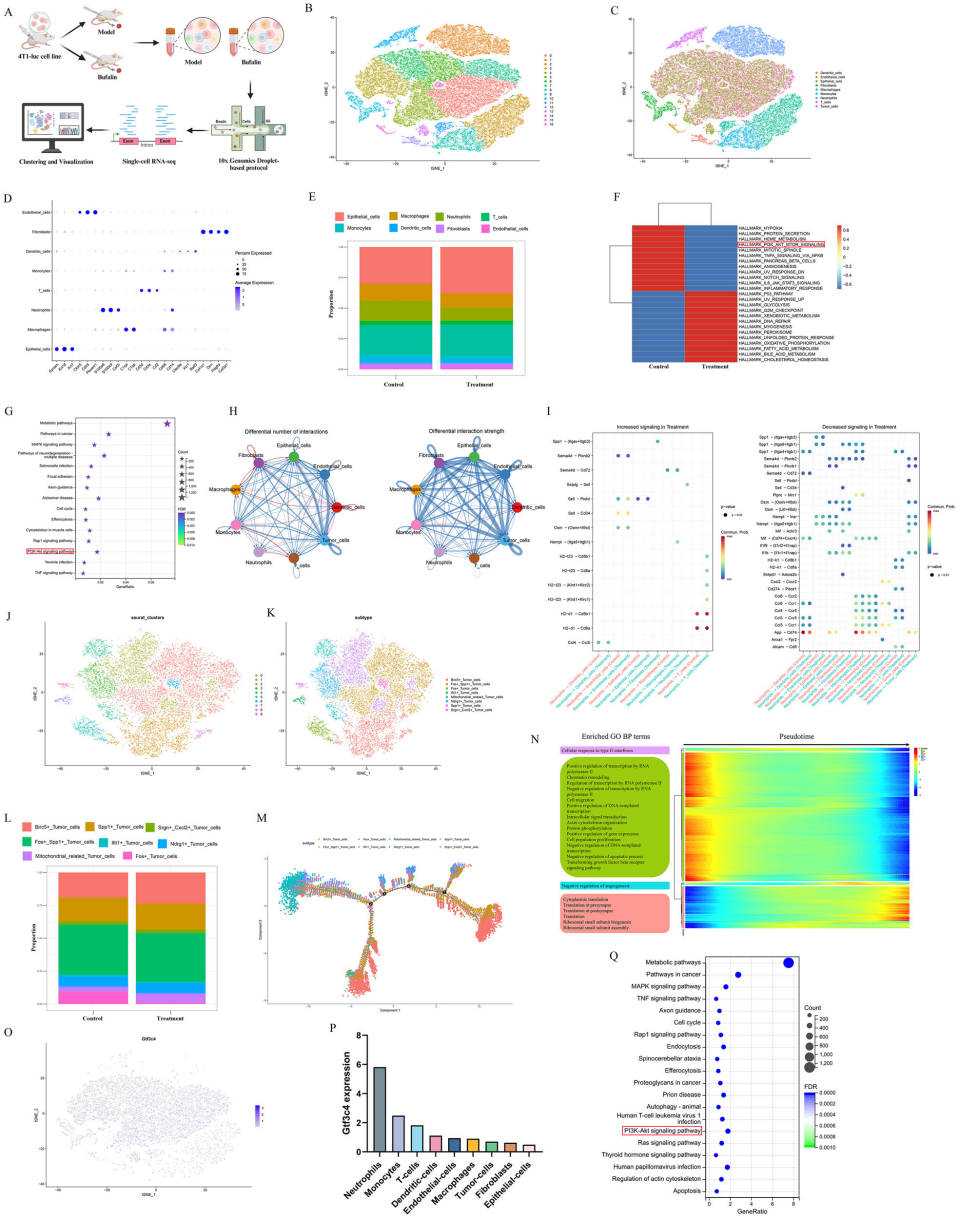
ScRNA‐seq was performed on tumors derived from the 4T1‐luc model. (A) The scRNA‐seq process. (B) Cell dimensionality reduction clustering analysis. (C) Cell annotation analysis. (D) Marker gene expression point map. (E) The proportions of different cell types in control and treatment groups. (F) GSVA enrichment analysis of the single‐cell data from different samples. (G) KEGG enrichment analysis. (H) CellChat analysis. (I) Ligand‐receptor mediated communication between neutrophils and various cell subtypes. (J) Tumor cells clustering. (K) Tumor cell annotation classification. (L) The proportions of tumor cell subtypes. (M) Pseudotime analysis was conducted on the eight tumor subtype cells. (N) Heatmap of dynamic pseudotime change of gene expression in tumor cells. (O) T‐SNE plot showing the expression distribution of Gtf3c4. (P) The expression of Gtf3c4 in different cell types. (Q) KEGG enrichment analysis of differentially expressed genes between Gtf3c4+ and Gtf3c4‐ groups.

While the anti‐tumor efficacy of bufalin and its effects on T cells and macrophages have been demonstrated in the 4T1‐luc model, its precise impact on the tumor microenvironment (TME) and the specific cell populations involved remain unclear. To address this, we performed scRNA‐seq to systematically characterize how bufalin reshapes the TME. Single‐cell suspensions prepared from tumor tissues were converted into single‐cell sequencing data using 10×Genomics. The scRNA‐seq process is shown in Figure 4A. Following quality control, both cell and gene libraries were generated for subsequent analysis. There were altogether 61,412 cells acquired in the data. T‐SNE was used to visualize the clustering situation, which was divided into 17 clusters with known or putative markers (Figure 4B). Based on the identified markers and CNV scoring, the cell populations were annotated, resulting in a total of nine cell types: epithelial cells, macrophages, neutrophils, T cells, monocytes, dendritic cells, fibroblasts, endothelial cells, and tumor cells (Figure 4C,D). The proportions of different cell types in control and treatment groups are illustrated in Figure 4E. The bufalin treatment group showed an increased proportion of epithelial cells, while the proportions of macrophages, neutrophils, and monocytes decreased. GSVA enrichment analysis of the single‐cell data from different samples revealed that bufalin treatment led to the suppression of the PI3K/AKT‐MTOR signaling pathway compared to the control group (Figure 4F). The PI3K/AKT signaling pathway was also obtained by KEGG enrichment analysis (Figure 4G). Through CellChat analysis of the samples, the cell communication network revealed the complex interactions between different cell types (Figure 4H). There was robust communication among macrophages, epithelial cells, fibroblasts, endothelial cells, and tumor cells. Figure 4I illustrates the probability of ligand‐receptor‐mediated communication between neutrophils and various cell subtypes. The ligand‐receptor between neutrophils and macrophages enhanced the signal of Nampt‐ (Itga5+Itgb1) in the bufalin administration group. The tumor cells were extracted for further dimensionality reduction and annotation analysis (Figure 4J). The top 10 marker genes with differential co‐expression from tumor cell subpopulations were selected for cell annotation and clustering, resulting in a total of eight cell types (Figure 4K). Birc5+ tumor cells and mitochondrial‐related tumor cells had a higher proportion in the bufalin treatment group, while Fos+ tumor cells had a lower proportion (Figure 4L). Pseudotime analysis was conducted on the eight tumor subtype cells (Figure 4M). The Ndrg1+ tumor cell subpopulation was primarily concentrated at the starting point of the trajectory, while the Birc5+ tumor cell subpopulation was mainly found at both the beginning and the end of the trajectory. In contrast, other subtypes are distributed throughout the entire pseudotime trajectory. As shown in Figure 4N, the genes that change with developmental time were divided into four clusters, each associated with transcriptional regulation by RNA polymerase II, cellular response to type II interferon, negative regulation of angiogenesis, and cytoplasmic translation, respectively. To investigate the expression of Gtf3c4 in cells, this study extracted data from single‐cell datasets for cells expressing Gtf3c4 and those not expressing Gtf3c4 and performed t‐SNE visualization (Figure 4O). Gtf3c4 expression was highest in neutrophils (Figure 4P). The KEGG enrichment analysis of differential genes between Gtf3c4+ and Gtf3c4‐ revealed enrichment of the PI3K/AKT signaling pathway (Figure 4Q). The signaling of various ligand‐receptor pairs between tumor cells and other cell types (T cells, neutrophils, monocytes, macrophages, fibroblasts, epithelial cells, endothelial cells, and dendritic cells) is weakened specifically in the Gtf3c4‐ group. Notably, pairs such as Col4a1‐Cd44, Angptl2‐(Itga5+Itgb1), and others show reduced signaling exclusively in the Gtf3c4‐ group, while some signals, like Sema4d‐Cd72, are weakened in both Gtf3c4+ and Gtf3c4‐ groups (Figure S3). Collectively, these findings elucidate how bufalin remodels the TME by modulating immune cell infiltration, oncogenic signaling, and cellular crosstalk.

### 2.5 GTF3C4 as a Key Risk Factor in Breast Cancer With Prognostic Biomarker Potential and Immune Interaction Significance

**FIGURE 5:**
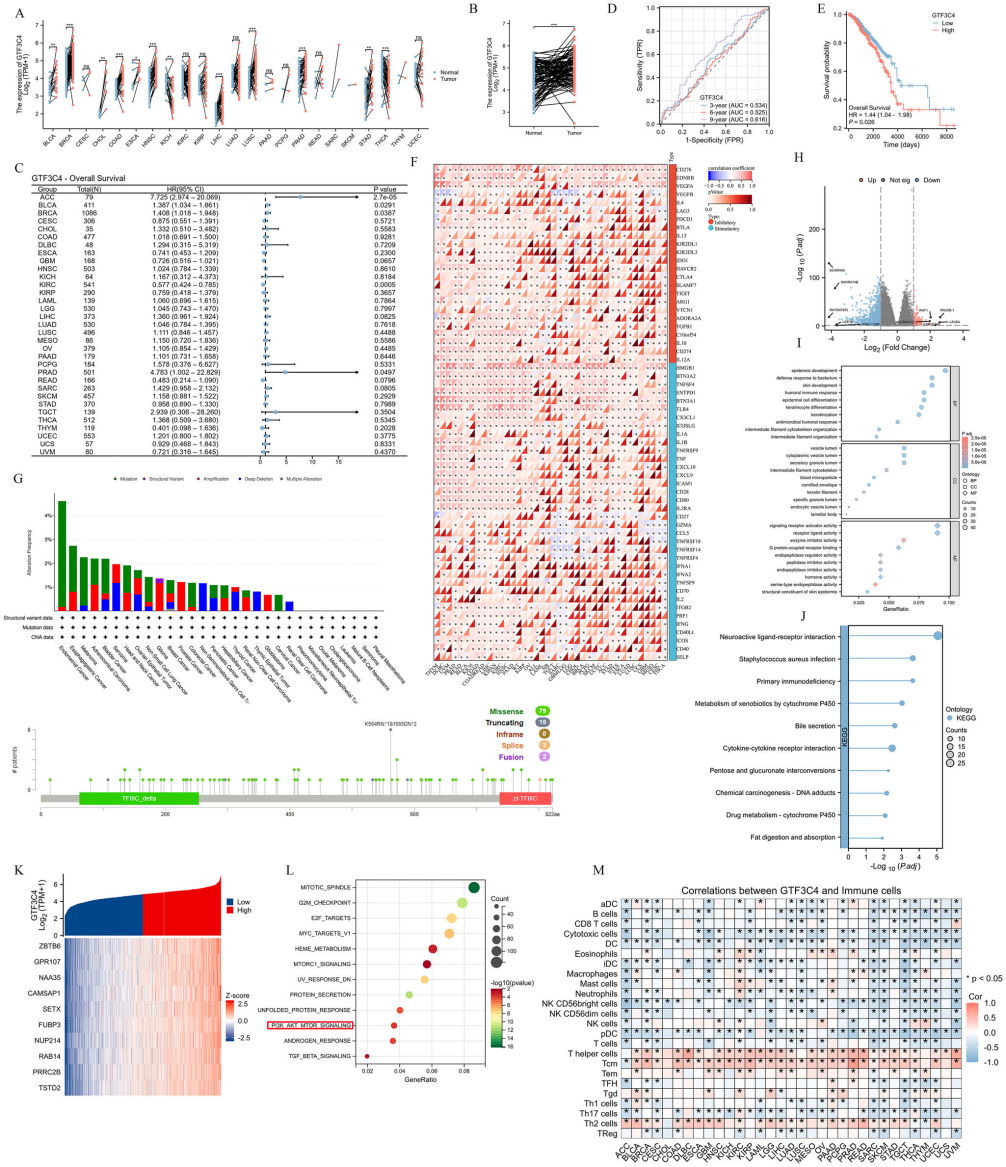
Bioinformatics analysis of the prognostic value of GTF3C4 in breast cancer. (A) The expression of GTF3C4 in paired cancerous and adjacent non‐cancerous tissues. (B) The expression of GTF3C4 in breast cancer. (C) Forest plot of Cox regression analysis of GTF3C4 expression and OS in cancer patients. (D) Time‐dependent ROC curve of GTF3C4 in breast cancer. (E) Kaplan‐Meier survival curve of GTF3C4 in breast cancer. (F) The correlation of GTF3C4 with immunoregulatory genes and immune checkpoint genes. (G) The correlation analysis of GTF3C4 and tumor gene mutation. (H) Volcano plot of differentially expressed genes between the GTF3C4 high‐expression group and low‐expression group. (I,J) GO and KEGG enrichment of differential genes. (K) Correlation analysis of GTF3C4 expression in breast cancer. (L) Hallmark analysis. (M) Correlations between GTF3C4 and immune cells. ^*^P < 0.05, ^**^P < 0.01, ^***^P < 0.001 and ns represented no statistical difference.

To systematically investigate the multifaceted role of GTF3C4 in tumorigenesis and progression, we conducted pan‐cancer analysis of the GTF3C4 gene revealed its expression patterns, prognostic correlations. GTF3C4 expression is significantly elevated in multiple tissues (including breast cancer) compared to adjacent non‐cancerous tissues (Figure 5A,B). To investigate the prognostic value of GTF3C4 in various cancers, this study established a Cox proportional hazards regression model to analyze the impact of GTF3C4 on overall survival (OS) in patients with breast cancer (Figure 5C). GTF3C4 has a certain prognostic value in breast cancer. The area under the curve (AUC) for predicting overall breast cancer survival was 0.534 at 3 years, 0.525 at 6 years, and 0.616 at 9 years (Figure 5D). As shown in Figure 5E, the OS of breast cancer patients with high expression of GTF3C4 was short (HR = 1.44, *p* = 0.026). High expression of GTF3C4 in breast cancer is associated with poor prognosis and is considered a risk factor. The correlation analysis of GTF3C4 expression with immune regulatory genes and immune checkpoint genes revealed that GTF3C4 expression is positively correlated with the majority of immune regulatory genes and immune checkpoint genes in breast cancer (Figure 5F). For breast cancer patients, the genetic changes are mainly Mutation, K564Rfs*18/1565Dfs*2 gene mutation frequency is high (Figure 5G). Based on the median expression levels of GTF3C4 in breast cancer patients from the TCGA database, classify the patients into a high‐expression group and a low‐expression group. As shown in Figure 5H, a total of 522 differential coding genes were screened, of which 30 differential genes were up‐regulated and 492 were down‐regulated. GO and KEGG enrichment analysis was performed on 522 differential genes (Figure 5I,J). In KEGG enrichment analysis, differential genes were mainly concentrated in the pathways of neural active ligand‐receptor interaction, cytochrome P450 metabolism of foreign substances, and cytokine‐cytokine receptor interaction. By performing a correlation analysis of GTF3C4 expression in breast cancer, we identified 5,721 genes based on the criteria of |Cor|>0.3 and *P*.adj <0.05. A heatmap was generated for the top 10 most highly correlated genes (ZBTB6, GPR107, NAA35, CAMSAP1, SETX, FUBP3, NUP214, RAB14, PRRC2B, and TSTD2) (Figure 5K). The items enriched by the Hallmark gene set are mainly related to the mitotic spindle, G2/M checkpoint, MYC target gene, and PI3K/AKT‐MTOR signaling pathway (Figure 5L). GTF3C4 is associated with a variety of immune cells in cancer, among which GTF3C4 is associated with neutrophils in breast cancer (Figure 5M). Our study revealed distinct expression patterns and significant prognostic correlations of GTF3C4 in breast cancer. Furthermore, genes associated with GTF3C4 expression in breast cancer are significantly enriched in the PI3K/AKT‐MTOR signaling pathway. These findings suggest that GTF3C4 may promote tumorigenesis and drive cancer progression through activation of the PI3K/AKT signaling cascade.

### 2.6 Bufalin Targeted GTF3C4 to Suppress the Proliferation of Breast Cancer

**FIGURE 6:**
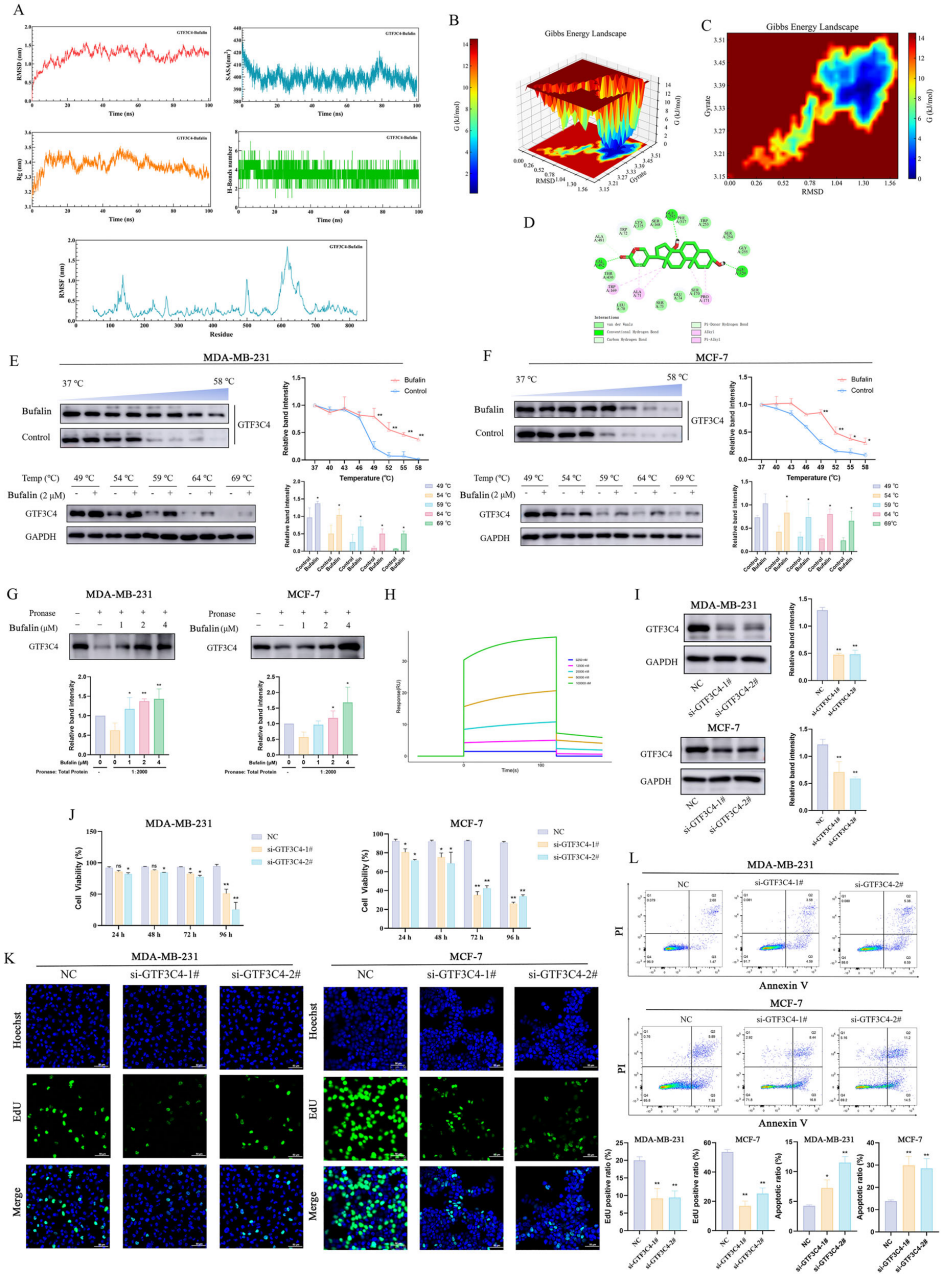
Bufalin targets GTF3C4 for the treatment of breast cancer. (A) Molecular dynamics simulation. (B, C) Gibbs free energy was visualized using both 3D and 2D contour plots. (D) The visual analysis of the GTF3C4‐Bufalin complex at the minimum Gibbs energy moment (89 ns). (E,F) Thermal stability of the GTF3C4 protein in MDA‐MB‐231 and MCF‐7 cells. (G) The effect of bufalin on GTF3C4 protein expression after pronase treatment in MDA‐MB‐231 and MCF‐7 cells. (H) Multi‐cycle kinetic test curve of bufalin and GTF3C4. (I) The expression of GTF3C4 in MDA‐MB‐231 and MCF‐7 cells after treatment with si‐GTF3C4‐1# and si‐GTF3C4‐2#. (J) Time‐dependent proliferation inhibition by GTF3C4 knockdown (24–96 h). (K) Representative images and statistical analysis of EdU staining (green) in MDA‐MB‐231 and MCF‐7 cells after treatment with si‐GTF3C4‐1# and si‐GTF3C4‐2#. (L) Flow cytometric analysis of apoptosis induction following GTF3C4 knockdown. (*n* = 3, ^*^P < 0.05, ^**^P < 0.01).

To further validate the interaction between GTF3C4 and bufalin, subsequent molecular dynamics simulation analysis was conducted. Molecular dynamics simulation analysis of the GTF3C4‐Bufalin complex system was carried out to study the dynamic properties of molecular docking (Figure 6A; Figure S4). The RMSD curve of the GTF3C4‐Bufalin complex shows that the system gradually reaches equilibrium after 80 ns, with an average RMSD of 1.3 nm. The Rg curve of the GTF3C4‐Bufalin complex shows a gradual decline in the system after 60 ns, with an average Rg of 3.36 nm. Based on the RMSD and Rg values of the GTF3C4‐Bufalin complex, the Gibbs free energy was calculated. The Gibbs free energy was then visualized in both 3D and 2D contour plots using the RMSD values, Rg values, and Gibbs free energy (Figure 6B,C). The Gibbs free energy landscape of the GTF3C4‐Bufalin complex is relatively rough, but there is still one distinct and sharp minimum energy region. The visual analysis of the GTF3C4‐Bufalin complex at the minimum Gibbs energy moment (89 ns) reveals that the GTF3C4‐Bufalin complex can form three hydrogen bonds (Figure 6D). Bufalin significantly enhanced the thermal stability of the GTF3C4 protein in MDA‐MB‐231 and MCF‐7 cells, indicating that bufalin can bind to the GTF3C4 protein in breast cancer cells (Figure 6E,F). The DARTS results demonstrated that bufalin could reverse the enzymatic hydrolysis of the GTF3C4 protein (Figure 6G). The binding affinity between GTF3C4 and bufalin was determined by surface plasmon resonance (SPR), with a measured KD value of 21.10 µm (Figure 6H). To functionally validate the oncogenic role of GTF3C4, we conducted GTF3C4 knockdown in MDA‐MB‐231 and MCF‐7 cells. As shown in Figure 6I, the levels of GTF3C4 significantly decreased in breast cancer cells after the knockdown of GTF3C4 by si‐GTF3C4‐1# and si‐GTF3C4‐2#. After GTF3C4 knockdown treatment in MDA‐MB‐231 and MCF‐7 cells, the proliferation of cells slowed down compared to the negative control (NC) group (Figure 6J). Furthermore, the inhibitory effect on cell proliferation became more pronounced with the extension of the knockdown duration (Figure 6J). As shown in Figure 6K, the EdU positive ratio in the si‐GTF3C4 group significantly decreased. In contrast, the cell apoptotic ratio increased after the knockdown of GTF3C4 (Figure 6L).

### 2.7 Bufalin Regulated the PI3K/AKT Signaling Pathway by Targeting GTF3C4

**FIGURE 7:**
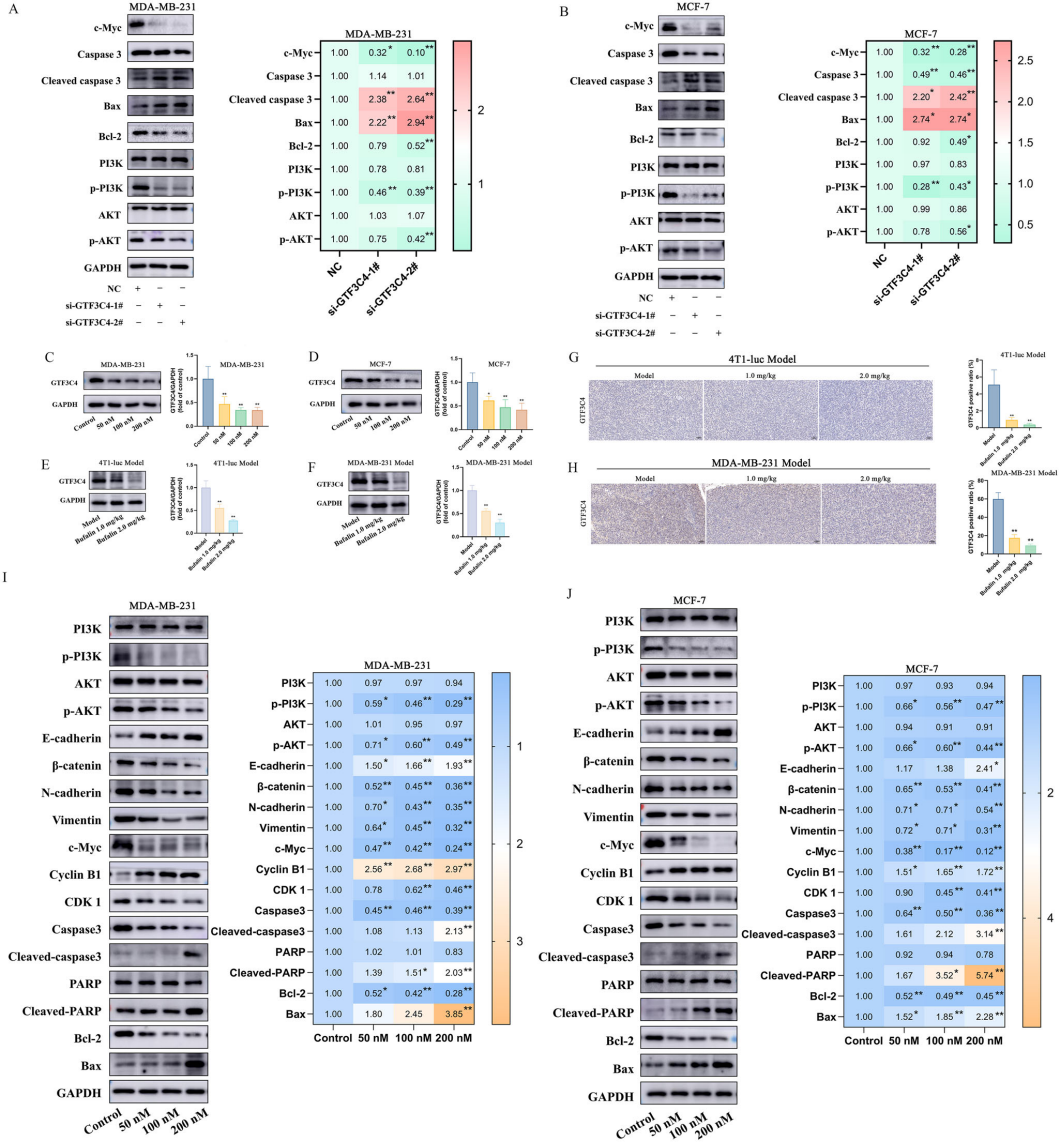
Bufalin regulated the PI3K/AKT signaling pathway by targeting GTF3C4. (A, B) The expression levels of c‐Myc, Caspase 3, Cleaved‐caspase3, Bax, Bcl‐2, PI3K, p‐PI3K, AKT, p‐AKT in MDA‐MB‐231 and MCF‐7 cells following knockdown of GTF3C4. (C,D) The expression of GTF3C4 in MDA‐MB‐231 and MCF‐7 cells following treatment with bufalin. (E,F) The expression of GTF3C4 in tumors from two animal models following treatment with bufalin. (G,H) Immunohistochemical staining of GTF3C4 on tumors in two animal models (*n* = 6). (I,J) The protein expression levels of PI3K, p‐PI3K, AKT, p‐AKT, E‐cadherin, β‐catenin, N‐cadherin, Vimentin, c‐Myc, Cyclin B1, CDK 1, Caspase 3, Cleaved‐caspase3, PARP, Cleaved‐PARP, Bcl‐2, Bax in MDA‐MB‐231 and MCF‐7 cells following treatment with bufalin. (*n* = 3, ^*^P < 0.05, ^**^P < 0.01).

Given the identification of GTF3C4 and PI3K/AKT signaling as critical targets for bufalin's anti‐breast cancer activity, we evaluated the effects of GTF3C4 knockdown on PI3K/AKT pathway regulation. Following the knockdown of GTF3C4, the expression levels of p‐PI3K, p‐AKT, Bcl‐2, and c‐Myc decreased in MDA‐MB‐231 and MCF‐7 cells (Figure 7A,B). In contrast, the expression levels of Bax and Cleaved‐caspase 3 increased (Figure 7A,B). As shown in Figure 7C,D, the expression of GTF3C4 was decreased in MDA‐MB‐231 and MCF‐7 cells after treatment with bufalin in a dose‐dependent manner. Western blot and immunohistochemistry results indicated that bufalin also reduced the expression of GTF3C4 on tumors in two animal models (Figure 7E–H). The mRNA expression levels of E‐cadherin, Bax, and Cyclin B1 significantly increased in MDA‐MB‐231 and MCF‐7 cells, while the mRNA expression levels of N‐cadherin, Vimentin, β‐catenin, c‐Myc, Bcl‐2, and CDK 1 significantly decreased after treatment with bufalin compared to the control group (Figure S5A,B). These results indicated that bufalin can inhibit the proliferation, migration, and invasion of breast cancer cells, induce apoptosis, and cause cell cycle arrest at the G2/M phase. In MDA‐MB‐231 and MCF‐7 cells, bufalin reduced the protein levels of β‐catenin, N‐cadherin, Vimentin, CDK 1, c‐Myc, Caspase3, Bcl‐2, p‐PI3K, and p‐AKT and increased the protein levels of E‐cadherin, Cyclin B1, Cleaved‐caspase3, Cleaved‐PARP and Bax (Figure 7I,J; Figure S6). In vivo studies demonstrated that bufalin potently suppressed the activation of PI3K/AKT signaling pathway in MDA‐MB‐231 breast cancer xenograft model (Figure S7). Bufalin treatment induced alterations in proteins associated with proliferation, migration, invasion, and apoptosis (Figure S7). Collectively, these results establish a mechanistic link between GTF3C4 knockdown, suppression of the PI3K/AKT signaling pathway, and bufalin‐mediated inhibition of breast cancer cell proliferation, migration, invasion, and induction of apoptosis, highlighting GTF3C4 and PI3K/AKT signaling pathway as key roles of bufalin's anti‐tumor efficacy.

## 3 Discussion

Breast cancer is one of the most common cancers among women, and its incidence continues to rise [4, 22]. In addition to surgery, radiotherapy, and chemotherapy, a variety of new treatment modalities are being explored [23, 24]. Bufalin possesses significant anti‐neoplastic activity against breast cancer, yet its complete mechanistic profile warrants systematic exploration. In this study, we systematically investigated how bufalin suppresses the malignant phenotypes of MDA‐MB‐231 and MCF‐7 cells. More importantly, the combined approach of LiP‐MS and RNA‐seq identified GTF3C4 and the PI3K/AKT signaling pathway as key molecular targets mediating bufalin's anti‐tumor effects. We further confirmed that bufalin binds to the GTF3C4 protein and suppresses the PI3K/AKT signaling pathway against breast cancer. In addition, we employed scRNA‐seq to systematically profile the cellular composition and molecular pathways altered by bufalin in the TME. Collectively, these findings not only elucidate the molecular mechanisms of bufalin but also highlight GTF3C4 and the PI3K/AKT pathway as potential therapeutic targets for breast cancer treatment.

GTF3C4 (TFIIIC90) is a subunit of the transcription factor TFIIIC complex and is mainly involved in the regulation of gene transcription mediated by RNA polymerase III. GTF3C4 was identified as one of the genes involved in cell senescence [25]. GTF3C4 helps in enzyme activation and DNA binding [26]. Long et al. [27]. utilized the data resources of TCGA to conduct a comprehensive analysis of the expression of histone acetylation regulatory genes in breast cancer and found that GTF3C4, as a histone acetylation writer, was closely related to the prognosis of breast cancer. Analysis of clinical transcriptomic samples from breast cancer patients revealed that the expression level of GTF3C4 is significantly upregulated in breast cancer tissues compared to normal breast tissue, and its high expression is closely associated with poor prognosis. Accumulated evidences suggest that the PI3K/AKT signaling pathway is widely activated in various cancers, and its importance as a therapeutic target has been confirmed by multiple studies [28]. The PI3K/AKT pathway plays a key role in the occurrence, development, and treatment of breast cancer [29]. This pathway promotes the formation and progression of tumors by regulating processes such as cell proliferation, apoptosis, metabolism and survival [30]. PI3K and AKT inhibitors have provided significant breakthroughs in the treatment of breast cancer [31, 32]. Some PI3K and AKT inhibitors have been used in clinical trials for the treatment of breast cancer, such as Buparlisib, Pictilisib, Taselisib, and Capivasertib [30, 32, 33, 34]. However, previous studies have not reported the association between GTF3C4 and the PI3K/AKT pathway in breast cancer. Based on the TCGA database, genes associated with GTF3C4 expression in breast cancer are significantly enriched in the PI3K/AKT‐MTOR signaling pathway. The PI3K/AKT signaling pathway was enriched with the most critical genes affected by bufalin. Following the knockdown of GTF3C4, the expression levels of p‐PI3K and p‐AKT decreased in MDA‐MB‐231 and MCF‐7 cells. Bufalin also inhibits the expression of GTF3C4 and the activation of PI3K/AKT signaling in MDA‐MB‐231, MCF‐7 cells, and tumor tissues. This study reveals the association between GTF3C4 and the PI3K/AKT pathway in breast cancer. In future studies, we will further explore the causal relationship between PI3K/AKT pathway inhibition and the observed phenotypic changes. Previous studies have shown that bufalin can promote apoptosis of cancer cells or inhibit their proliferation, invasion, and metastasis by regulating various signaling pathways, such as the PI3K/AKT pathway, MAPK‐JNK pathway, and Wnt‐β‐catenin pathway [13, 35, 36]. Our findings illuminate that bufalin inhibited the activation of the PI3K/AKT signaling pathway by targeting GTF3C4. Furthermore, bufalin regulates EMT‐related proteins, modulates the cell cycle, and affects apoptosis‐related proteins to suppress the proliferation, migration, and invasion of breast cancer cells while promoting their apoptosis.

More and more evidence showed that the immunosuppressive TME is one of the reasons for the failure of immunotherapy response, and the composition of immune cells in TME plays a key role in regulating immunosuppressive properties, which can induce tumor proliferation and recurrence, and affect the efficacy of immunotherapy [37, 38, 39, 40]. The advancement of scRNA‐seq technology has greatly facilitated the investigation of the TME. Macrophages play an important role in the tumor microenvironment [41]. M1 and M2 macrophages serve as a double‐edged sword in cancer therapy [42]. M1 macrophages, also known as classically activated macrophages, are a crucial force in anti‐tumor immune responses, directly or indirectly killing tumor cells. In contrast, M2 macrophages exhibit pro‐tumor effects in the tumor microenvironment. It is worth noting that bufalin affects macrophages in the immune microenvironment of colorectal cancer and hepatocellular carcinoma [43, 44, 45]. Bufalin promoted the polarization of tumor‐infiltrating macrophages to the M1 phenotype in hepatocellular carcinoma, thereby stimulating anti‐tumor immune responses and affecting the tumor microenvironment [43]. Our findings revealed that there was an increase in M1 macrophages and a decrease in M2 macrophages in both tumor and spleen tissues. It is hypothesized that the anti‐breast cancer effects of bufalin may be partially attributed to its influence on macrophage polarization. By promoting the polarization of M1 macrophages and inhibiting the polarization of M2 macrophages, bufalin may enhance the anti‐tumor immune response. Utilizing scRNA‐seq, our results indicated bufalin alters the TME in the transplanted tumor. The bufalin treatment group showed an increased proportion of epithelial cells, while the proportions of macrophages, neutrophils, and monocytes decreased. Macrophages, neutrophils, and monocytes in the tumor microenvironment typically promote inflammation and tumor progression [46, 47, 48, 49, 50]. GSVA enrichment analysis of single‐cell data from different samples revealed that the PI3K/AKT‐MTOR signaling pathway was inhibited following bufalin administration compared to the control group. Additionally, the PI3K/AKT signaling pathway was identified through KEGG enrichment analysis. This finding is consistent with the key PI3K/AKT signaling pathway identified in the RNA‐seq analysis and bioinformatics analysis. The tumor cells were extracted and analyzed again for dimension reduction annotation. Compared with the model group, Fos+ Tumor cells accounted for a lower proportion of cells in the bufalin administration group. The Fos family (including c‐Fos, FosB, FosL1, and FosL2) plays a significant role in the occurrence and progression of various malignancies [51, 52, 53, 54]. Bufalin may act on Fos+ tumor cells, thereby affecting the proliferation and metastasis of tumor cells, which leads to an anti‐tumor effect. Notably, GTF3C4 is highly expressed in neutrophils, and its expression can influence the strength of receptor‐ligand signaling communication between different cells. The KEGG pathway of differential genes of GTF3C4+ and GTF3C4‐ cell subsets were also involved in the PI3K/AKT signaling pathway. Although bufalin has shown good potential in the treatment of breast cancer, there are still some limitations. The current research has not yet included cell co‐culture experiments to verify the effect of bufalin on the interaction between tumor cells and immune cells. Furthermore, the efficacy of bufalin in different subtypes of breast cancer still requires further research. Future research should combine multi‐omics techniques and animal models to comprehensively evaluate the anti‐tumor mechanism and clinical application prospects of bufalin.

## 4 Conclusion

In summary, this study provides a systematic description of the mechanism by which bufalin inhibits breast cancer. We present evidence for the efficacy of bufalin in the treatment of breast cancer both in vivo and in vitro. By employing LiP‐MS, RNA‐seq, and scRNA‐seq, bufalin can target GTF3C4 to inhibit the PI3K/AKT pathway and remodel the tumor microenvironment, thereby hindering the malignant progression of breast cancer. Overall, our study explores the mechanism of action of bufalin against breast cancer, providing valuable insights into its potential therapeutic applications.

## 5 Experimental Section

### 5.1 Reagents

Bufalin was purchased from Selleck (Batch: S782101). Cell Counting Kit (CCK‐8) and EdU Cell Proliferation Kit were obtained from Biorigin Co., Ltd (Beijing, China). Crystal violet dye was purchased from Biosharp Co., Ltd (Beijing, China). Cell cycle Analysis Kit was purchased from Beyotime Biotechnology Co., Ltd (Shanghai, China). Cell apoptosis assay kit was supplied by BD Biosciences (San Jose, CA, USA). The total RNA extraction kit was purchased from Tiangen Biotech Co., Ltd (Beijing, China). ReverTra Ace qPCR RT Kit and SYBR Green Realtime PCR Master mix were obtained from Toyobo Co., Ltd (Osaka Prefecture, Japan). Lipofectamine 2000 transfection reagent was purchased from Thermo Fisher Scientific (Waltham, MA, USA).

### 5.2 Cell lines

MDA‐MB‐231, MCF‐7, and MCF‐10A were purchased from Wuhan Pricella Biotechnology Co., Ltd (Wuhan, China). 4T1‐luc was purchased from Shanghai Fuheng Biotechnology Co., Ltd (Shanghai, China). MDA‐MB‐231 and MCF‐7 cells were cultured in DMEM and MEM medium containing 10% Fetal Bovine Serum (FBS) and 1% Penicillin‐Streptomycin (PS), respectively. MCF‐10A was cultured in DMEM/F12 medium with 5% HS, 20 ng/mL EGF, 0.5 µg/mL Hydrocortisone, 10 µg/mL Insulin, 1% NEAA, and 1% PS. 4T1‐luc was cultured in RPMI 1640 medium containing 10% FBS and 1% PS. All cells were cultured in an incubator at 37°C in an atmosphere of 5% CO_2_.

### 5.3 Colony Formation

MDA‐MB‐231 and MCF‐7 cells were planted in a 6‐well plate at a density of 500 cells/well. After 3 days, the medium was replaced with bufalin‐medium which was changed every three days for each group. On day 12, cells were rinsed with PBS, and a 4% tissue fix solution was added. Then, cells were stained with 0.1% crystal violet solution.

### 5.4 EdU Cell Proliferation Assay

The experiment was carried out according to EdU Cell Proliferation Kit instructions. In brief, 2×10^5^ cells were plated in a confocal dish. After 24 h, the cells were cultured with various concentrations of bufalin for 48 h. After EdU reagent was added for 2 h, the cells were fixed with 4% paraformaldehyde for 15 min. Fluorescence images were captured using a confocal laser scanning microscope after staining with Hoechst 33342 for 20 min in the dark.

### 5.5 Wound Healing Assay

MDA‐MB‐231 and MCF‐7 cells were planted in a 6‐well plate at a density of 8 × 10^5^ cells/well for 24 h. A standard 200 µL sterile pipette was employed to create deliberate wounds on the confluent cell layer. Subsequently, cells were exposed to a medium containing 0, 50, 100, and 200 nm bufalin. The wound was observed and captured under the inverted microscope after 0, 24, and 48 h. Image J 1.8 was used to measure the scratch area.

### 5.6 Transwell Assay

In the migration assay, cells were resuspended in a medium containing 0.2% BSA with or without bufalin. 5×10^4^ cells were added into the upper chamber of a 24‐well transwell plate with an 8 µm pore filter (Corning Inc., USA). The lower was added 600 µL serum‐free medium (DMEM or MEM) containing 10% FBS. Following a 24 h incubation, the cells were fixed with 4% paraformaldehyde and stained with crystal violet. The effect of bufalin on cell migration was observed under an inverted microscope. In the invasion assay, the matrigengel, chambers, and 24‐well plates are pre‐cooled at 4°C first. Then, 60 µL matrigengel was added to the upper chamber and placed in the incubator at 37°C overnight. The other operations are the same as the migration experiment.

### 5.7 Cell Cycle Analysis

MDA‐MB‐231 and MCF‐7 cells were seeded in a 6‐well plate at a density of 5×10^5^ cells/well and incubated for 24 h. After exposure to varying concentrations of bufalin for 48 h, the cells were harvested, fixed in 70% ethanol at 4°C overnight, and then centrifuged. The cells were incubated with a staining buffer at 37°C for 30 min in the dark, and cell cycle progression was assessed using flow cytometry (Beckman, USA).

### 5.8 Cell Apoptosis Assay

MDA‐MB‐231 and MCF‐7 cells were seeded in a 6‐well plate at a density of 4 × 10^5^ cells/well for 24 h. After exposure to different concentrations of bufalin for 48 h, cells were harvested and stained with 5 µL of Annexin V‐FITC and PI. Then, cells were instantly detected by flow cytometry (Beckman, USA).

### 5.9 Reactive Oxygen Species

MDA‐MB‐231 and MCF‐7 cells were incubated with bufalin for 48 h. Both cells were harvested and incubated with DCFH‐DA for 20 min. After incubation, the cells were washed three times with DMEM or NEM medium. Then, fluorescence intensity was measured by flow cytometry (Beckman, USA).

### 5.10 Animal Experiments

Female BALB/c mice (5–6 weeks old, 18 ± 2 g) and Female BALB/c‐nu nude mice (5–6 weeks old, 15 ± 2 g) were purchased from SPF (Beijing) Biotechnology Co., Ltd. All animal procedures comply with China's Regulations on the Administration of Experimental Animals, and have been approved by the Animal Experiment Ethics Committee of Beijing University of Chinese Medicine (Ethics Approval Number: BUCM‐2023101003‐4025 and BUCM‐2024052404‐2131). All the mice were housed in the Specific Pathogen‐Free (SPF) animal facility at the Laboratory Animal Center of Beijing University of Chinese Medicine. To establish a transplanted tumor model of breast cancer, female BALB/c mice were injected with 4T1‐luc cells at a density of 2 × 10^6^ cells/mL. Female BALB/c‐nu nude mice were injected with MDA‐MB‐231 cells at a density of 5 × 10^7^ cells/mL. Tumor length (a) and width (b) were measured, and tumor volume (V) was calculated using the formula V (mm^3^) = πab^2^/6. Treatment with bufalin commenced when tumor volume reached 50–100 mm^3^. In 4T1‐luc model, the mice were randomly divided into five groups (*n* = 6): Model, Bufalin (0.5 mg/kg), Bufalin (1.0 mg/kg), Bufalin (2.0 mg/kg), and Paclitaxel (10.0 mg/kg). In MDA‐MB‐231 model, the mice were randomly divided into four groups (*n* = 6): Model, Bufalin (1.0 mg/kg), Bufalin (2.0 mg/kg), and Paclitaxel (10.0 mg/kg). Bufalin was administered intraperitoneally every 2 days, while paclitaxel was administered every 3 days. The growth of tumors in mice was observed by a small animal imaging system. Flow cytometry was used to analyze the tumor tissue and spleen of the mice. Cell suspension of tumors and spleen were obtained by rapid and gentle stripping, physical grinding, and filter filtration. Cells were stained with APC anti‐mouse CD45, Alexa Fluor 700 anti‐mouse CD3, Brilliant Violet 510 anti‐mouse CD4, Brilliant Violet 650 anti‐mouse CD8a, APC/Cyanine7 anti‐mouse/human CD11b, PE/Cyanine7 anti‐mouse F4/80, and PE anti‐mouse CD86. After being treated with fixation buffer and permeabilization buffer, intracellular CD206 was stained using FITC anti‐mouse CD206 (MMR). Stained cells were analyzed by flow cytometry (Beckman, USA). Alanine aminotransferase (ALT), aspartate aminotransferase (AST), urea nitrogen (BUN), uric acid (UA), lactate dehydrogenase (LDH‐L) and creatine kinase (CK) were detected to assess of liver function, renal function, and myocardial enzyme markers. The experiment is based on the corresponding kit instructions. TUNEL staining kit (Servicebio, Wuhan) was used for investigating the number of TUNEL‐positive cells in tumors.

### 5.11 Hematoxylin‐Eosin (H&E) Staining and Immunohistochemical Staining

The tissues were immersed in 4% paraformaldehyde for 48 h. Subsequently, the tissues were embedded in paraffin and sliced into 5 µm tissue slices. For general histology, the sections were stained with hematoxylin and eosin (Servicebio, Wuhan). For immunohistochemistry, the sections were treated with 3% hydrogen peroxide before being incubated with specific primary antibodies against Ki67 (1:600), PCNA (1:1000), MMP9 (1:500), CD86 (1:200), CD206 (1:400) and GTF3C4 (1:200). The slices were then stained with DAB chromogenic solution and incubated with the corresponding secondary antibody for 2 h, followed by counterstaining with hematoxylin.

### 5.12 Limited Proteolysis‐Mass Spectrometry (LiP‐MS)

The MDA‐MB‐231 cell lysate was mixed with different concentrations of bufalin, and subjected to limited proteolysis with proteinase K (PK). After adding denaturant (UA/DOC) and DTT, incubate the sample at 30°C for 2 h. Next, add IAA, shake at 600 rpm for 1 min, and protect from light for 30 min. Dilute the UA/DOC concentration by adding NH_4_HCO_3_ buffer. Then, add 2 µg of Trypsin and incubate at 37°C for 16 h. After desalting and lyophilization, redissolve the peptides in 0.1% FA and measure the concentration at OD_280_. Take 100 µg of low‐abundance peptides separated from high‐abundance peptides and perform fractionation using the HPRP method, collecting all components. After lyophilizing each component, redissolve in 10 µL of 0.1% FA and measure the peptide concentration at OD_280_. Then, take 2 µg of each peptide, add an appropriate amount of iRT standard peptide, and perform DIA mass spectrometry analysis. HPLC system EasynLC was used for the separation. The separated samples were analyzed by DIA mass spectrometry using a Q‐Exactive HFX instrument. Spectronaut (Spectronaut TM 14.4.200727.47784) was used for data processing.

### 5.13 RNA sequencing

Total RNA was extracted from MCF‐10A cells, as well as from MDA‐MB‐231 and MCF‐7 cells following exposure to bufalin (MCF‐7‐L and MCF‐7‐H: treated with 100 and 200 nm bufalin; MDA‐MB‐231‐L and MDA‐MB‐231‐H: treated with 100 nM and 200 nM bufalin). After confirming that the samples met the quality standards, mRNA was enriched using magnetic beads linked with Oligo(dT) to capture eukaryotic mRNA. Using the mRNA as a template, the first strand of cDNA was synthesized with random hexamer primers, followed by the addition of buffer, dNTPs, and DNA Polymerase I to synthesize the second strand of cDNA. The resulting double‐stranded cDNA was then purified using AMPure XP beads. The purified double‐stranded cDNA underwent end repair, A‐tailing, and ligation of sequencing adapters. Fragment size selection was performed using AMPure XP beads, and PCR enrichment was carried out to obtain the final cDNA library. Once the library passed the quality check, different libraries were pooled according to the target sequencing data amount, and sequencing was performed. RNA sequencing was carried out by Shanghai Applied Protein Technology Co., Ltd. The HISAT2 software was used to align clean reads to the specified genome, providing information about their locations on the reference genome. The FPKM values for each gene's expression in each sample were calculated using featureCounts software. The R package DESeq2 was employed to analyze the differential expression of genes across different groups. The criteria for identifying differentially expressed genes were set as |logFC|>1 and *P.adj*<0.05. ClusterProfiler was utilized to annotate genes with Gene Ontology (GO) terms. Additionally, the Kyoto Encyclopedia of Genes and Genomes (KEGG) enrichment analysis of differentially expressed genes was carried out using the “KEGG.db” and “compareCluster” packages in R software.

### 5.14 Single‐Cell RNA Sequencing

Based on the expected number of captured cells, GEMs (Gel Beads in Emulsion) are constructed for single‐cell separation. The GEMs were treated to break the oil phase, and one‐strand cDNA was purified and enriched using magnetic beads. Subsequently, cDNA amplification and quality control were performed. The qualified cDNA was then used to construct a 3' end gene expression library. The library was quantitatively detected using the Qubit Assay Tube. Finally, sequencing was performed on the Illumina NovaSeq 6000 platform.

### 5.15 ScRNA‐Seq Quality Control and Data Processing

The original sequencing data were analyzed using FastQC software. Data quality statistics for the raw FastQ data were generated using Cell Ranger software. Based on the expression matrix generated by Cell Ranger, the Seurat package was used for cell filtering. The NormalizeData function was used to normalize (standardize) all single‐cell data. The RunTSNE function was used to perform t‐distributed Stochastic Neighbor Embedding (t‐SNE) for dimensionality reduction of the single‐cell data, and the DimPlot function was used for t‐SNE visualization. The FindAllMarkers function was used to identify marker genes. The infercnv package was used for copy number variation (CNV) analysis, annotating tumor cells based on CNV scores. Gene set database information was obtained from the msigdbr package, selecting “Mus musculus” as the organism and the Hallmark gene sets. Subsequently, the GSVA package was used to perform enrichment analysis. GO and KEGG enrichment analyses were performed on the differentially expressed genes (DEGs). The communication probabilities were calculated using the computeCommunProb function, followed by filtering the communication data with the filterCommunication function. The aggregateNet function was then used to compute the aggregated intercellular communication network. The monocle package was used for pseudo‐time series analysis of tumor cells.

### 5.16 Bioinformatics Analysis of the Prognostic Value of GTF3C4 in Breast Cancer and Pan Carcinoma

RNA‐seq data for 33 cancer types, including breast cancer, was downloaded from the TCGA database (https://portal.gdc.cancer.gov). The expression of GTF3C4 across breast cancer and the other 32 cancer types was analyzed using the ggplot2, stats, and car packages. The survival package was utilized to test the proportional hazards assumption and conduct a Cox regression analysis. Time‐dependent ROC data analysis was performed on the data using the timeROC package. The Kaplan‐Meier survival curve was constructed to predict the prognosis with high/low expression of GTF3C4. Pearson correlation analysis was conducted between GTF3C4 and associated immune data. Gene mutation information for GTF3C4 in breast cancer and other tumors was explored using the cBioPortal for Cancer Genomics (http://www.cbioportal.org/), assessing mutation frequency, mutation sites, and copy number variations. Breast cancer patients were divided into high‐ and low‐expression groups based on the median expression level of GTF3C4. Differential expression analysis was performed on the two groups using the DESeq2 package in R, with filtering criteria of |log2 FC|>1 and *P* < 0.05. Furthermore, GO and KEGG enrichment analyses were conducted on the differentially expressed genes to explore GTF3C4‐related biological functions.

### 5.17 Molecular Docking and Molecular Dynamics Simulation (MD)

In this study, AutoDock Vina v1.2.3 software was used for molecular docking analysis. The docking was performed to calculate the binding affinity (kcal/mol). The details of the ligand‐receptor interactions were analyzed and visualized using PyMOL v2.6 and Discovery Studio (DS) 2021 Client software, generating both 3D and 2D interaction diagrams. Molecular dynamics simulations of the complexes obtained from molecular docking were performed using Gromacs software for 100 ns. Based on the results of the MD, the root‐means‐square deviation (RMSD), root‐mean‐square fluctuation (RMSF), radius of gyration (Rg), solvent‐accessible surface area (SASA), and hydrogen bonds (H‐bonds) were calculated. Using the RMSD and Rg values, the Gibbs free energy was calculated with the built‐in “g_sham” and “xpm2txt.py” scripts of Gromacs. Furthermore, the binding free energy of the complex was calculated using the molecular mechanics/Poisson‐Boltzmann surface area (MM/PBSA) method with the “MMPBSA.py v.16.0” script.

### 5.18 Cellular Thermal Shift Assay (CETSA)

MDA‐MB‐231 and MCF‐7 cells were inoculated into 10 cm dishes for 24 h. Then cells were treated with bufalin (2 µm) or DMSO for 2 h. After treatment with 0.25% pancreatic enzyme (including EDTA), the solutions were heated at the indicated temperatures (37°C, 40°C, 43°C, 46°C, 49°C, 52°C, 55°C, 58°C or 49°C, 54°C, 59°C, 64°C, 69°C) for 3 min. After repeated freeze‐thaw cycles, and centrifuge at 4°C for 15 min at 10 000 g, the soluble supernatant was subject to a western blot.

### 5.19 Drug Affinity Responsive Target Stability (DARTS)

The cells were treated with pre‐chilled M‐PER buffer containing phosphatase and protease inhibitors and placed on ice for lysis for 10 min. The supernatant was collected after centrifugation at 12 000 g for 15 min at 4°C. The protein concentration in the lysate was regulated with a pre‐cooled 1×TNC buffer to an optimal concentration of 4–6 µg/µL. Then, to allow sufficient ligand‐protein target binding, samples were gently mixed followed by incubation for 2 h at room temperature. The protease liquor (10 mg/mL) was diluted by 1×TNC to 0.2 µg/µL. The aliquoted lysates were then digested with protease at a 1:2000 ratio (protease to total protein concentration) for exactly 30 min. Next, a loading buffer was added to the lysates, and the mixture was boiled for 10 min.

### 5.20 Surface Plasmon Resonance (SPR)

SPR was utilized to assess the differences in affinity between the GTF3C4 and bufalin, as well as evaluate their binding and dissociation kinetic characteristics. NTA chip was used in this experiment. Different concentrations of bufalin (6250, 12500, 25000, 50000, 100000 nm) were flowed over the blank sensor and the target sensor with GTF3C4 at a flow rate of 30 µL/min, followed by a 120‐second binding and a 60‐second dissociation phase. The binding and dissociation constants were determined using the BIAcore T200 software, which globally fit the data to a 1:1 Langmuir binding model.

### 5.21 RNA Interference and Transfection

Complementary oligonucleotide sequences of siRNAs were designed and synthesized by GenePharma (Shanghai, China). These siRNA or NC vectors were transfected into MDA‐MB‐231 and MCF‐7 cells by lipofectamine 2000. Three siRNA sequences of GTF3C4 were listed in Table S1.

### 5.22 Quantitative Reverse‐Transcriptase Polymerase Chain Reaction (qRT‐PCR)

Total RNA was extracted from cells using the Total RNA extraction kit from Tiangen Biotech (Beijing) Co., Ltd. Equal amounts of total RNA (1 µg) were subjected to reverse transcription using the ReverTra Ace qPCR RT Kit. For RT‐qPCR, SYBR Green Real‐time PCR Master Mix (Toyobo, Japan) was utilized, with GAPDH serving as the reference gene. The primer sequences were listed in Table S2.

### 5.23 Western Blot

Cells or tissue samples were kept on ice and lysed using RIPA buffer that included protease and phosphatase inhibitors. The supernatant was obtained by centrifuging at 12 000 rpm for 20 min at 4°C, and protein concentrations were determined with the BCA Detection Kit (Servicebio, Wuhan, China). The protein samples were separated using SDS‐PAGE and subsequently transferred onto a nitrocellulose (NC) membrane. The membranes were blocked with 5% skim milk for 2 h. Following this, the primary antibody specific to the target protein was added and incubated overnight at 4°C. Then, the membranes were then treated with a secondary antibody for 1.5 h at room temperature. An imaging system was used to visualize the protein bands, and Image J 1.8 was employed for quantifying protein expression. The antibodies were used in the supplementary material.

### 5.24 Statistical Analysis

Data were statistically analyzed by GraphPad Prism 9.5.1. The measurement data involved are expressed as mean ± standard deviation ($\bar{x}$±S.D.). The differences between two groups were analyzed by two‐tailed unpaired Student's t‐test, and the differences among three or more groups were analyzed by one‐way variance (ANOVA) and Tukey's honest significant difference (Tukey's HSD) test. *P *< 0.05 indicated that the difference was statistically significant.

## Author Contributions

S.G. and X.C. contributed equally to this work. Writing – original draft, Conceptualization, Investigation, Formal analysis, Data curation, S.G., X.C.; Data curation, Validation, H.W., J.Z., P.L., and J.L.; Validation, K.C., J.Z., S.Y., S.L., Y.G., Z.J., and X.T.; Conceptualization, Supervision, Validation, Z.H.; Conceptualization, Supervision, Validation, Q.L.; Conceptualization, Supervision, Project administration, Funding acquisition, J.W. All authors approved the final manuscript.

## Conflicts of Interest

The authors declare no conflicts of interest.

## Supporting information




**Supporting file**: advs74313‐sup‐0001‐SuppMat.docx

## Data Availability

The data that support the findings of this study are available from the corresponding author upon reasonable request.
