# Supplementary material for: Bufalin Inhibits the PI3K/AKT Pathway by Targeting GTF3C4 to Impede Breast Cancer Progression
Source: Adv Sci (Weinh). 2026 Feb 6;13(23):e07008. doi: 10.1002/advs.202507008 (PMC13104141; doi:10.1002/advs.202507008)
Supplement: Supplementary file 1 — Supporting file: advs74313‐sup‐0001‐SuppMat.docx [file ADVS-13-e07008-s001.docx]

**Bufalin Inhibits the PI3K/AKT Pathway by Targeting GTF3C4 to Impede Breast Cancer Progression**

*Siyu Guo^1†^, Xiaodong Chen^1†^, Haojia Wang^1^, Jiying Zhou^1^, Peiying Lu^1^, Jiangying Liu^1^, Keyan Chai^1^, Jingyuan Zhang^1^, Siyun Yang^1^, Shan Lu^1^, Yifei Gao^1^, Zhengsen Jin^1^, Xiaoyu Tao^1^, Zhongdong Hu^2,*^,* *Qinglin Li^3,*^, Jiarui Wu**^1,*^*

^1^Department of Clinical Chinese Pharmacy, School of Chinese Materia Medica, Beijing University of Chinese Medicine, Beijing 102488, China

^2^Modern Research Center for Traditional Chinese Medicine, Beijing Institute of Traditional Chinese Medicine, Beijing University of Chinese Medicine, Beijing 100029, China

^3^Zhejiang Cancer Hospital, Hangzhou Institute of Medicine (HIM), Chinese Academy of Sciences, Hangzhou, Zhejiang 310022, China

*^†^* These authors contributed equally to this work.

*^*^*Address correspondence to:

Zhongdong Hu, email: [zdhu@bucm.edu.cn](mailto:exogamy@163.com) ；

Qinglin Li, email: [qinglin200886@126.com](mailto:exogamy@163.com)；

Jiarui Wu, email: [exogamy@163.com](mailto:exogamy@163.com)

**Contents**

**Materials and Methods**

Quantitative Reverse-Transcriptase Polymerase Chain Reaction (qRT-PCR)

Table S1. Three siRNA sequences of GTF3C4

| Number | Name | Sequence (5’- 3’) |
| --- | --- | --- |
| 1# | GTF3C4-Homo-1088 | Sense Strand: GGUGGGAAUAUGAGCACAATT  Antisense Strand: UUGUGCUCAUAUUCCCACCTT |
| 2# | GTF3C4-Homo-2130 | Sense Strand: GGAAGAGAAACUCCUGGAATT  Antisense Strand: UUCCAGGAGUUUCUCUUCCTT |
| 3# | GTF3C4-Homo-999 | Sense Strand: GCAGUUUCAGCUGCCGUUUTT  Antisense Strand: AAACGGCAGCUGAAACUGCTT |

Table S2. Primer sequences used in real-time PCR.

| Primers | Primer sequence（5’-3’） |
| --- | --- |
| GTF3C4 | Forward: CTCCTCAAAGTTGGCTCAAAAAC |
|  | Reverse: AACATGAAAGTCTGACTGACCG |
| E-cadherin | Forward: ATTTTTCCCTCGACACCCGAT  Reverse: TCCCAGGCGTAGACCAAGA |
| N-cadherin | Forward: AGCCAACCTTAACTGAGGAGT  Reverse: GGCAAGTTGATTGGAGGGATG |
| Vimentin | Forward: ACCAAGACCTGCTCAATGTTAAGATG  Reverse: TCCTGCTCTCCTCGCCTTCC |
| β-catenin | Forward: AGAGGCTCTTGTGCGTACTGTC  Reverse: TTGGTGTCGGCTGGTCAGATG |
| c-Myc | Forward: CCTGGTGCTCCATGAGGAGAC  Reverse: CAGACTCTGACCTTTTGCCAGG |
| Bax | Forward: ACTCCCCCCGAGAGGTCTT  Reverse: CAAAAGTAGAAAAGGGCCGACAA |
| Bcl-2 | Forward: ATGTGTGTGGAGAGCGTCAACC  Reverse: TGAGCAGAGTCTTCAGAGACAGC |
| Cyclin B1 | Forward: GCCAGTGCCAGAGCCAGAAC  Reverse: GCCAGTGCCAGAGCCAGAAC |
| CDK 1 | Forward: TGCCGCTCTCCACCATCCG  Reverse: GCACACATCAAACAACCTGACCAC |
| GAPDH | Forward: TGGAGTCCACTGGCGTCTTCAC  Reverse: TTGCTGATGATCTTGAGGCTGTTGTC |

**Western Blot**

The following antibodies were used: anti- Cleaved Caspase 3 (Affinity, OH, USA), anti-Caspase 3 (Proteintech, Wuhan, China), PARP (Affinity, OH, USA), anti-Cleaved PARP (Affinity, OH, USA), anti-Bax (ABclonal, Wuhan, China), anti-Bcl2 (Abclonal, Wuhan, China), anti-CDK1 (ABclonal, Wuhan, China), anti-CyclinB1 (ABclonal, Wuhan, China), anti-E-cadherin (Proteintech, Wuhan, China), anti-β-catenin (Proteintech, Wuhan, China), anti-N-cadherin (Proteintech, Wuhan, China), anti-Vimentin (Proteintech, Wuhan, China), anti-GTF3C4 (Proteintech, Wuhan, China), anti-P-PI3K (Affinity, OH, USA), anti-PI3K(Proteintech, Wuhan, China), anti-P-AKT(Proteintech, Wuhan, China), anti-AKT (Proteintech, Wuhan, China), anti-c-Myc (Proteintech, Wuhan, China), anti-iNOS (ABclonal, Wuhan, China), anti-Arg-1 (Selleck, Shanghai, China) and anti-GAPDH (Proteintech, Wuhan, China).

**Figures**


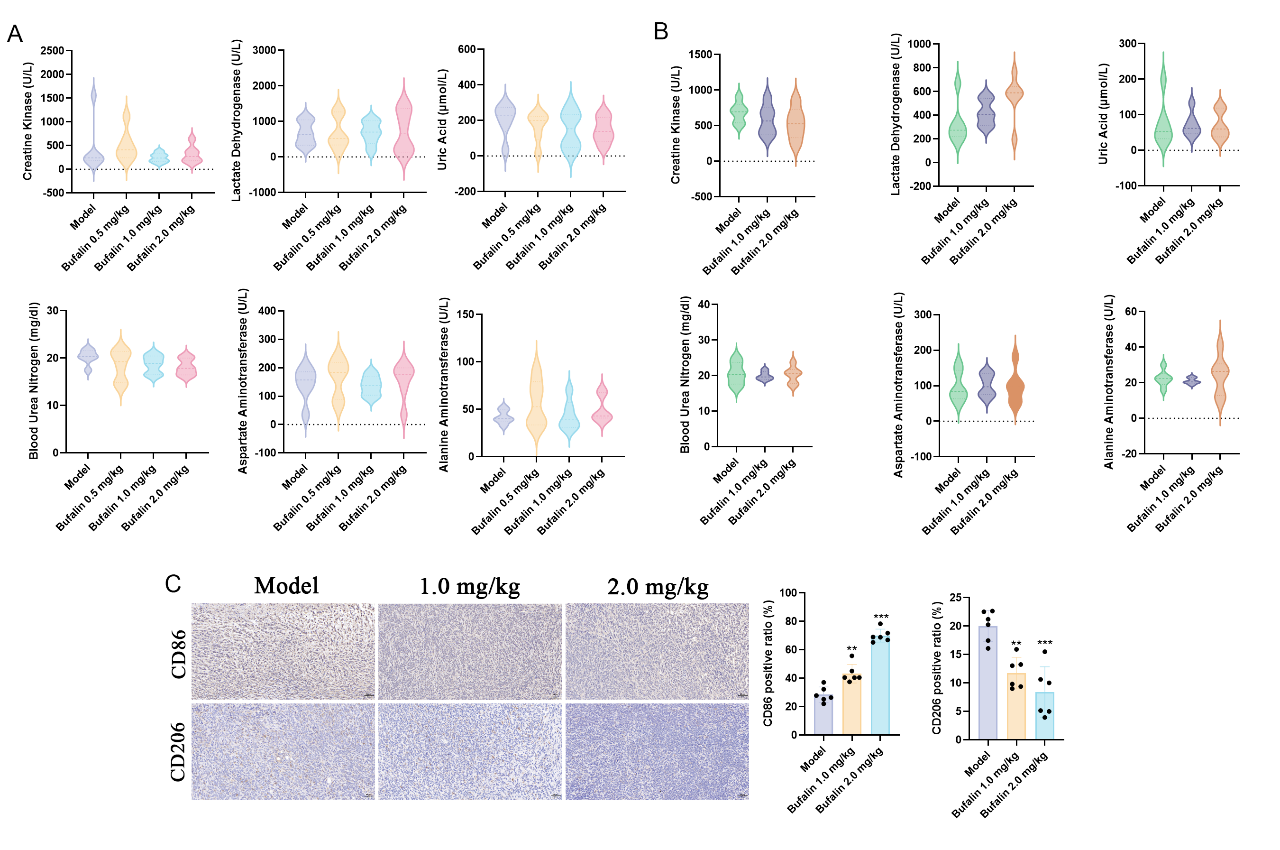


Figure S1. Alanine aminotransferase (ALT), aspartate aminotransferase (AST), blood urea nitrogen (BUN), uric acid (UA), lactate dehydrogenase (LDH), and creatine kinase (CK) levels in 4T1-luc model (A) and MDA-MB-231 model (B) (n=6). (C) Representative images of IHC staining of CD86 and CD206.


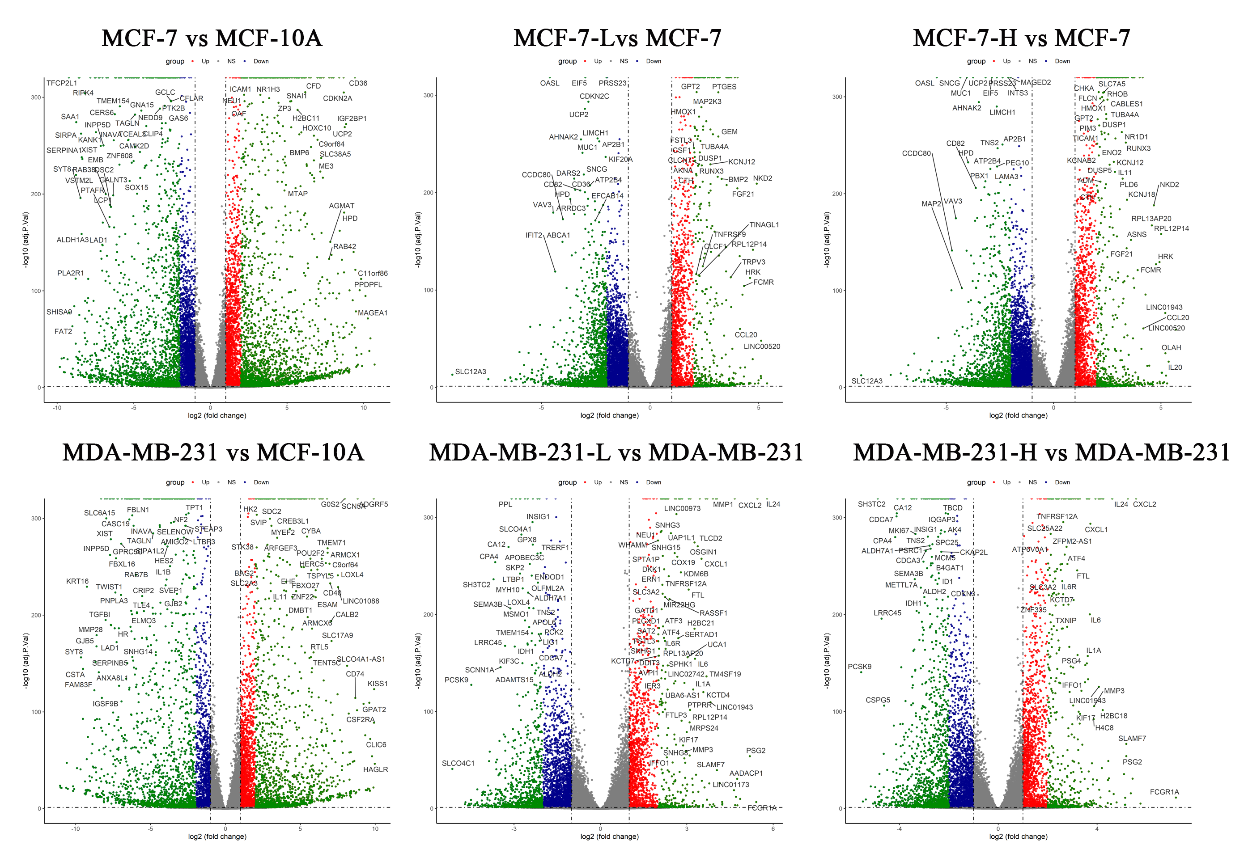


Figure S2. Volcano plot of differentially expressed genes (MCF-7 vs. MCF-10A, MCF-7-L vs. MCF-7, MCF-7-H vs. MCF-7, MDA-MB-231 vs. MCF-10A, MDA-MB-231-L vs. MDA-MB-231, and MDA-MB-231-H vs. MDA-MB-231).


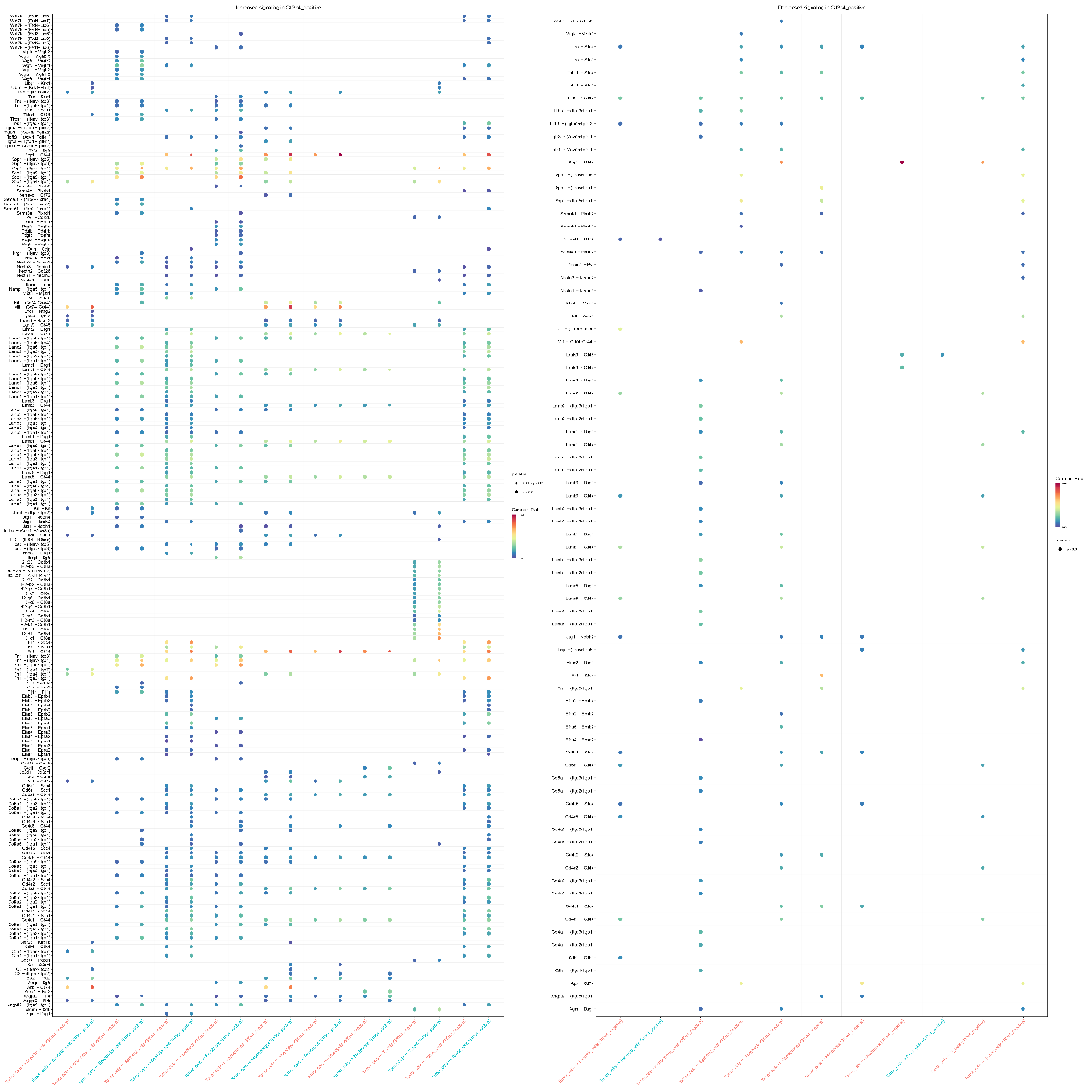


Figure S3. Probability of ligand-receptor pin-mediated communication between tumor cells (Gtf3c4) and different cell subtypes.


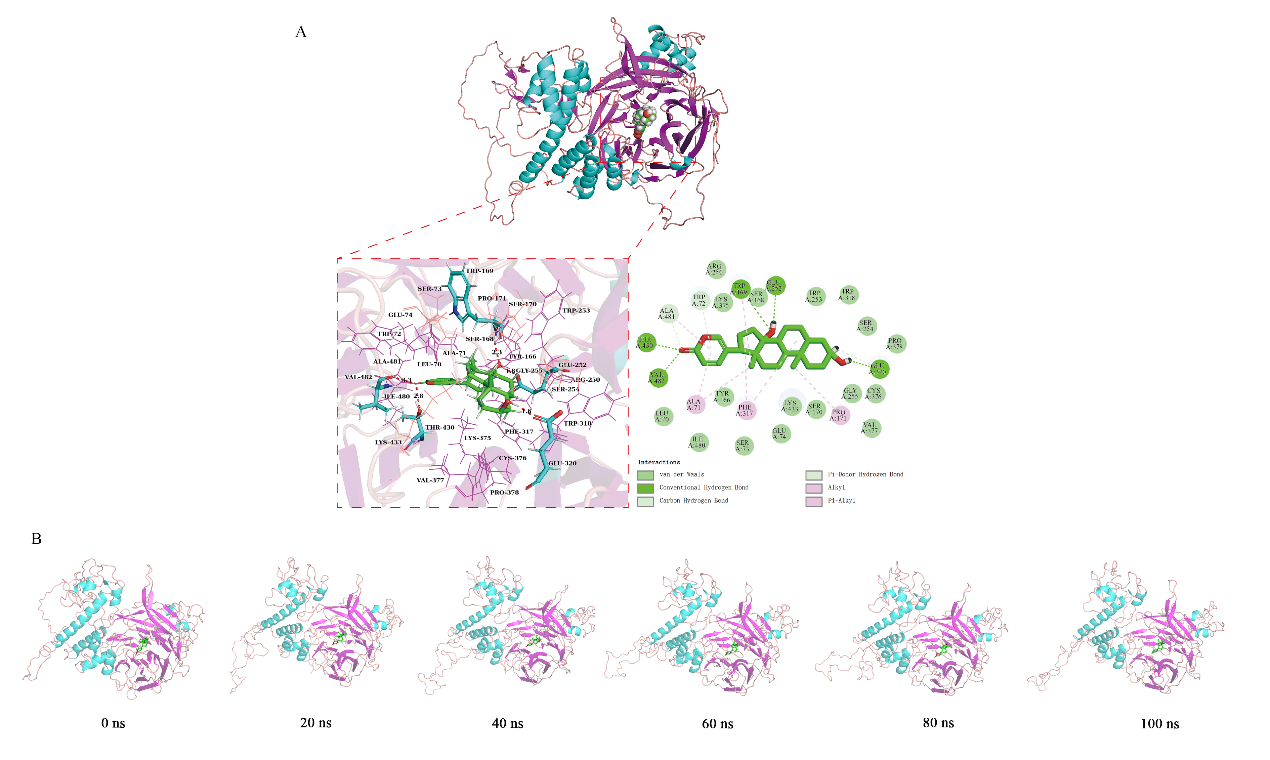


Figure S4. (A) Analysis of GTF3C4 docking with Bufalin. (B) 100 ns molecular dynamics simulation analysis of GTF3C4-Bufalin complex system.


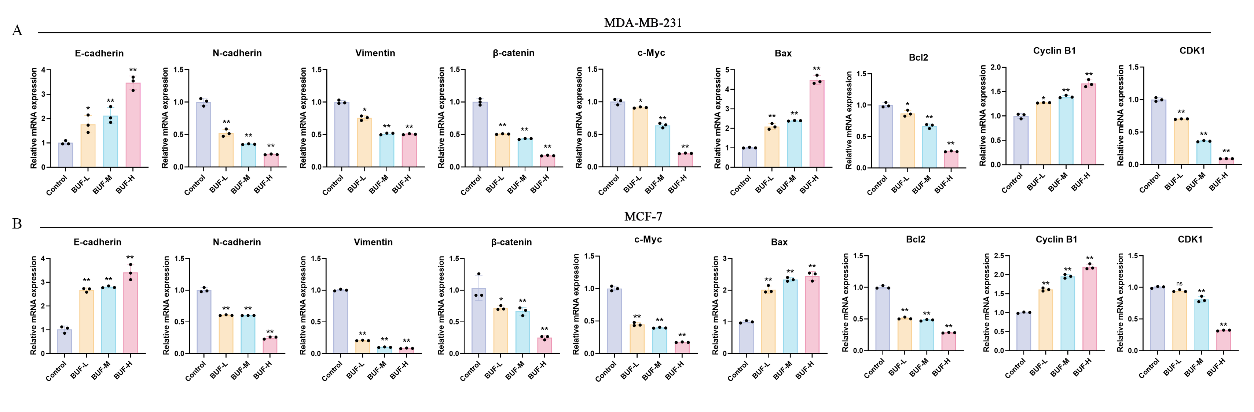


Figure S5. The mRNA expression levels of E-cadherin, N-cadherin, Vimentin, β-catenin, c-Myc, Bax, Bcl-2, Cyclin B1 and CDK 1 in MDA-MB-231 and MCF-7 cells. BUF-L, BUF-M, and BUF-H represent bufalin-50 nM, -100 nM and -200 nM, respectively. (n=3, **P*<0.05, ***P*<0.01).


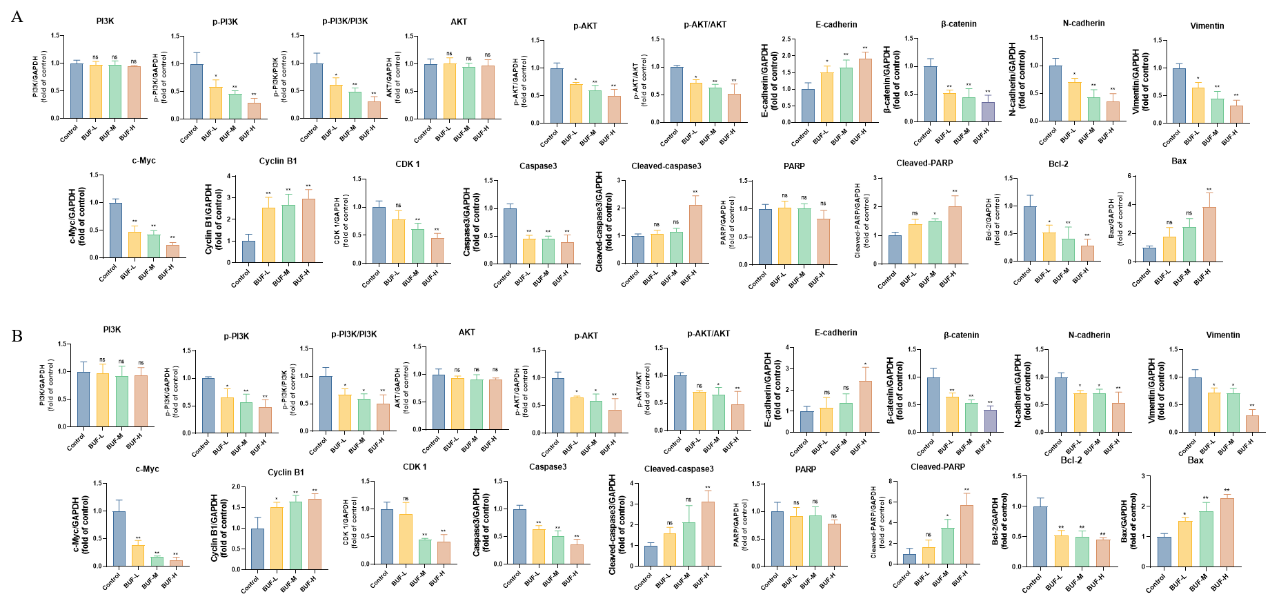


Figure S6. The quantitative graph of protein expression in MDA-MB-231 and MCF-7 cells. BUF-L, BUF-M, and BUF-H represent bufalin-50 nM, -100 nM and -200 nM, respectively. (n=3, **P*<0.05, ***P*<0.01 and ns represented no statistical difference).


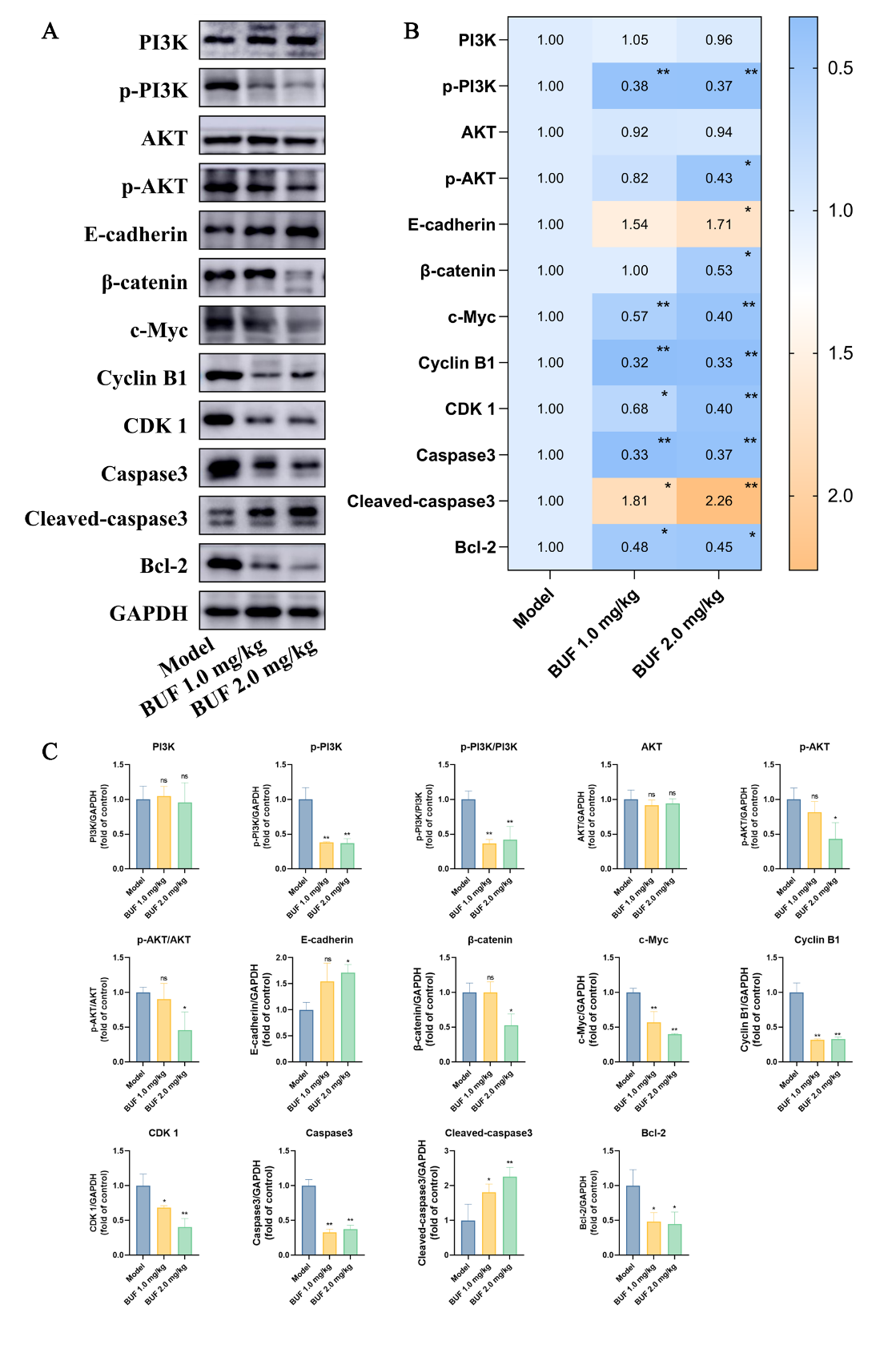


Figure S7. The protein expression levels of PI3K, p-PI3K, AKT, p-AKT, E-cadherin, β-catenin, c-Myc, Cyclin B1, CDK 1, Caspase 3, Cleaved-caspase3, Bcl-2 in tumor tissue following treatment with bufalin. (n=3, **P*<0.05, ***P*<0.01 and ns represented no statistical difference).
